# Axonal structure-function relationships across experimental modalities

**DOI:** 10.1162/IMAG.a.1058

**Published:** 2025-12-22

**Authors:** Christian S. Skoven, Mariam Andersson, Miren Lur Barquin Torre, Marco Pizzolato, Hartwig R. Siebner, Tim B. Dyrby

**Affiliations:** Danish Research Centre for Magnetic Resonance, Department for Radiology and Nuclear Medicine, Copenhagen University Hospital Amager and Hvidovre, Copenhagen, Denmark; Center of Functionally Integrative Neuroscience, Department of Clinical Medicine, Aarhus University, Aarhus, Denmark; Department of Applied Mathematics and Computer Science, Technical University of Denmark, Kongens Lyngby, Denmark; Department of Neurology, Copenhagen University Hospital Bispebjerg and Frederiksberg, Copenhagen, Denmark; Department of Clinical Medicine, Faculty of Medical and Health Sciences, University of Copenhagen, Copenhagen, Denmark

**Keywords:** corpus callosum, conduction velocity, axon diameter, rat, motor cortex

## Abstract

The structure-function relationship of myelinated nerve fibers (Waxman & Bennett, 1972) links axon diameter and myelin thickness, commonly expressed as the g-ratio, to conduction velocity via saltatory mechanisms. Here, we investigated this relationship in the transcallosal motor pathway of the rat brain by combining functional and structural metrics in the same animals. Transcallosal conduction times (TCTs) were measured using local field potentials (LFPs) evoked by optogenetic stimulation of excitatory neurons in the motor cortex. Conduction velocity estimates were obtained by combining TCTs with transcallosal tract lengths derived from diffusion MRI (dMRI)-based tractography. Fluorescent labeling of the viral optogenetic construct verified the tractography trajectories. In parallel, axon diameter and g-ratio were quantified using both dMRI and transmission electron microscopy (TEM). To assess dehydration-induced tissue shrinkage associated with conventional TEM preparation (“Epon-TEM”), we also performed cryo-fixation followed by TEM (“Cryo-TEM”) in a separate group. This revealed diameter-dependent axonal shrinkage, yielding a correction factor of 37%. Shrinkage correction improved agreement between dMRI and Epon-TEM estimates, although dMRI remained biased toward larger axons. When translated via the structure-function relationship, TCTs corresponded to smaller axons near the mode of the TEM diameter distribution, while dMRI-based diameters predicted TCTs that were too short compared with the recorded LFP latencies. Altogether, our findings show that structural and functional metrics differ in their sensitivity profiles. Accounting for such modality-dependent sensitivities facilitates the investigation of structure-function relationships, advancing our understanding of how microstructure supports neural communication.

## 1 Introduction

The microstructural morphology of axons forming the long-distance white matter tracts between functional brain regions modulates saltatory conduction velocity and, consequently, the timing of brain function. Rushton (1951) proposed a theoretical relationship between axon diameter, myelin thickness, and conduction velocity, drawing on experimental data from Hursh (1939), Sanders and Whitteridge (1946), among others. Waxman and Bennett (1972) revised this structure-function relationship to better account for the conduction velocities of small-diameter axons in the central nervous system (CNS). A key parameter in this relationship is the ratio of the inner axon diameter to total outer diameter including the myelin sheath—the “g-ratio” which simulation studies predict to be optimally around 0.7 (Chomiak & Hu, 2009; Tettoni et al., 1996), consistent with histological studies showing relatively constant g-ratio in CNS axons across species (Innocenti et al., 2014; Stikov et al., 2015; Waxman & Bennett, 1972; Waxman & Swadlow, 1976). Assuming cylindrical geometry and a fixed g-ratio, conduction velocity is directly proportional to axonal diameter (Waxman & Bennett, 1972). Although other physiological factors also influence conduction velocity (Arancibia-Cárcamo et al., 2017; Drakesmith et al., 2019; Hursh, 1939; Rushton, 1951), axon diameter and myelin remain its primary determinants (Drakesmith et al., 2019).

The distribution of axon diameters across white matter tracts depends on the functional roles of the brain regions they interconnect (Innocenti et al., 2014). This is exemplified by the corpus callosum (CC), which contains most of the interhemispheric fibers in mammals (Aboitiz et al., 2003; Assaf et al., 2020; Owen, 1837) and has been consistently shown through light microscopy (LM) and electron microscopy (EM) to exhibit varying axon diameter distributions across the midsagittal plane, with the largest diameters associated with motor and somatosensory areas (Aboitiz et al., 1992; Caminiti et al., 2009; Riise & Pakkenberg, 2011; Tomasi et al., 2012). By combining measurements of tract length obtained via neuronal tracers or diffusion magnetic resonance imaging (dMRI) tractography with LM measurements of axon diameter distribution and assumptions of constant g-ratio, Tomasi et al. (2012) and Caminiti et al. (2013) predicted conduction latencies between connected regions in monkeys. These predictions, which used the relation between conduction velocity and axon outer diameter from Waxman and Bennett (1972), closely match electrophysiological measurements and stimulation experiments in other animal studies (Caminiti et al., 2013; Swadlow et al., 1978).

*In vivo* estimation of axon diameter can be obtained with dMRI, which exploits the restricted diffusion of water molecules to non-invasively probe the geometry of axons (Alexander et al., 2010; Dyrby et al., 2018; Fan et al., 2020; Veraart et al., 2020). These estimates rely on fitting a biophysical model to the measured dMRI signal (Alexander et al., 2010; Andersson et al., 2022; Barazany et al., 2009; Fan et al., 2020; Pizzolato et al., 2023; Veraart et al., 2020) and represent a voxel-averaged metric of axonal morphology, rather than direct measurements of individual axons, as each MRI voxel (typically ~1 mm³) encompasses thousands of axons. Horowitz et al. estimated the axon diameter distribution *in vivo* in humans and correlated the mean diameter index with conduction velocities obtained from electroencephalography (Horowitz et al., 2015). However, dMRI tends to overestimate axon diameters compared to histological measurements (Alexander et al., 2010; Barazany et al., 2009; Dyrby et al., 2018; Fan et al., 2020; Veraart et al., 2020), and the upper and lower bounds (Dyrby et al., 2013; Nilsson et al., 2017) of measurable axon diameter are constrained by the MRI hardware and imaging protocol (Andersson et al., 2022; Dyrby et al., 2013; Nilsson et al., 2017; Sepehrband et al., 2016). Importantly, smaller axons often fall below typical lower bounds. Moreover, unlike the simple arithmetic mean of the diameter distribution used in 2D histology from LM or EM, dMRI-based axon diameter estimates are biased toward large diameters due to the volumetric nature of MRI and the scaling of signal attenuation with axon size (Alexander et al., 2010; Burcaw et al., 2015; Lee et al., 2020; Packer & Rees, 1972; Veraart et al., 2020).

Although smaller axons can be measured with LM and EM, traditional tissue preparation typically involves a dehydration step prior to embedding in resin. This causes shrinkage of the tissue up to 65% (Aboitiz et al., 1992; Dyrby et al., 2018). In contrast, cryo-fixation better preserves the native tissue structure, thus providing measurements of axon diameter closer to the hydrated *in vivo* state (Korogod et al., 2015).

This study aims to use the structure-function relationship, described by Hursh (1939) and others (Waxman & Bennett, 1972) in rat CNS transcallosal axons to relate structural and functional measurements from the same animals. Specifically, we estimated axon diameters from dMRI, and measured axon diameters/g-ratios from transmission electron microscopy (TEM) with classical tissue processing (“Epon-TEM”). We examine how these structural measures relate to electrophysiological recordings of conduction velocity along a transcallosal pathway. To assess shrinkage from dehydration in classical tissue processing, we performed cryo-fixation before TEM (“Cryo-TEM”) in an additional rat cohort and compared the resulting axon diameter distributions to those measured with Epon-TEM.

## 2 Materials and Methods

All animal procedures were conducted in accordance with the ARRIVE guidelines, the European Communities Council Directive (2010/63/EU), and were approved by the Animal Experiments Inspectorate (2016-15-0201-00868) of Denmark. The electrophysiological raw data were collected as part of our previous study (Skoven et al., 2022), where detailed methods can be found. The data were reanalyzed in the present study to obtain the transcallosal conduction time (TCT).

### 2.1 Animals and surgery for optogenetics and electrophysiology

Sixteen young (4 weeks old) male Sprague-Dawley rats (NTac:SD-M) underwent stereotaxic surgery. Bilateral craniotomies were performed over the M1 primary motor cortices (AP: +1.0 mm; ML: ±2.5 mm, relative to bregma). In the right M1, the viral inoculum (AAV5::CaMKIIα−hChR2(H134R)−EYFP; UNC Vector core) was injected at -1.00 mm depth relative to the dura, followed by implantation of a calibrated optic fiber (Ø = 55 µm). In the left M1, a stainless steel stereotrode pair (Ø = 127 µm per electrode) was implanted at the same depth to record the evoked potentials as LFPs.

### 2.2 Electrophysiological recording of optogenetically evoked transcallosal potentials

The animals were exposed to laser light stimulation (λ = 447 nm) via the optical fiber while recording the evoked responses as LFPs during dexmedetomidine and low dose isoflurane anesthesia (Østergaard et al., 2022). For peak detection, the signals from the two channels of the stereotrode were averaged for each trial and all trials were averaged and baseline corrected. The first positive peak (P1) and negative peak (N1) were automatically detected within manually determined time spans (based on visual inspection): t = + 1 ms to t = + 9 ms for P1, and t = + 5 ms to t = + 20 ms for N1. Data from one animal (rat33.1) were discarded due to lacking response after optogenetic stimulation. The coefficient of variation was calculated as the standard deviation (SD) of the signal amplitude of all individual trials at the peak latency, divided by the mean signal amplitude at the peak latency. A condition was considered reliable when the coefficient of variation of the trials for the N1 peak, being the most pronounced of the two, was less than 1.00. One animal (rat9.4, in addition to rat33.1, mentioned above) produced no reliable conditions based on this criterion, and was excluded. The TCT was represented by the latencies of the first measurable transcallosal response (P1). Data from one additional animal (rat13.2), producing no detectable P1, were excluded. An overview of the included TCTs (N = 13) and included/excluded data, in general, can be seen from Supplementary Table S1 and Supplementary Table S5, respectively.

### 2.3 Perfusion fixation

Immediately following the final electrophysiology experiment, rats were transcardially perfused with 0.1 M potassium phosphate-buffered saline (KPBS) delivered at 15 mL/min for 3 min, followed by 7–12 min of perfusion with 4% formaldehyde in PBS. The brains were extracted and post-fixed at 4°C for at least 3 weeks.

### 2.4 Post mortem MRI

#### 2.4.1 Preparation prior to MRI scan

Due to long scan protocols (in total 44 h), only a subset of the brains (N = 9) was prepared for MRI scanning. At least 3 weeks before the MR scans, the brains were placed in 0.1 M KPBS (Dyrby et al., 2018). Each was scanned in a double-lined plastic bag containing a minimal volume of room temperature KPBS, on a sample holder custom-made with LEGO^TM^ (Dyrby et al., 2011). The samples were scanned in a cryo-coil setup on a 7T preclinical Bruker scanner (Bruker BioSpec 70/20 USR) using Paravision 6.0.1.

#### 2.4.2 Diffusion MRI

For axon diameter estimation, we used a three-shell pulsed gradient spin echo (PGSE) protocol with single-line readout. Three unique b-values with b = [24450, 21247, 17560] s/mm^2^, gradient strengths G = [590, 550, 500] mT/m, gradient separation (∆) = 18 ms, and gradient duration (∂) = 8 ms were applied along 30 uniformly distributed diffusion gradient directions (Bak & Nielsen, 1997; Jones et al., 1999). In addition, nine b = 0 images were acquired. All shells used the same echo time (TE) of 28.5 ms and repetition time (TR) of 3500 ms and were acquired with an isotropic voxel size of 125 µm. Ten sagittal slices with no slice gap covered a limited field of view around the midsagittal region of the CC. Total acquisition time was 35 h.

For tractography, whole-brain dMRI data sets with isotropic voxel size of 125 µm; 80 axial slices; no slice gap; matrix: 128 × 128 × 80; FOV: 16 mm × 16 mm × 10 mm were collected. We used a single shell with b-value of 4000 s/mm^2^ (gradient strength (G) = 224 mT/m; ∆ = 23 ms; ∂ = 7.5 ms) in 61 isotropic distributed non-collinear directions (Cook et al., 2006). The scan was acquired with TE = 38.6 ms and TR = 3592 ms. Total acquisition time was 9 h.

The dMRI data sets were denoised using the variance stabilizing transform and optimal shrinkage singular value manipulation method (Ma et al., 2020), and processed to remove Gibbs ringing artifacts (Kellner et al., 2016) using the MRTrix3 software toolbox (Tournier et al., 2019).

#### 2.4.3 High resolution structural image

A high-resolution structural 3D T2-weighted MR image was obtained for N = 5 rats for determination of implant depths. A “true” fast imaging with steady-state free precession (FISP) T2 weighted sequence acquired whole-brain visualization with the following parameters: TR: 2.5 s; TE: 5.1 ms; matrix size: 256 × 256 × 128; field of view: 23.04 × 23.04 × 11.52 mm^3^; image resolution: 90 × 90 × 90 µm^3^; flip angle: 30 degrees; averages: 40; and total acquisition time: 2 h.

#### 2.4.4 Tractography

We performed probabilistic streamline tractography on the whole-brain dMRI data set (N = 9). Generating a combined bilateral tractogram, with seed and target in each respective hemisphere, was not feasible. Instead, the most robust way to segment the tract was to sum the lengths of two individual tractograms generated separately for each hemisphere. One tractogram emanated from the optical fiber depth and the other from the electrode depth, both set to terminate in the midsagittal CC. All ROIs were manually drawn on fractional anisotropy (FA) maps generated using the MRtrix3 toolbox (Tournier et al., 2019). The seed ROIs for each hemisphere were drawn axially, just beneath the shaft lesion from fiber or electrode implantation, while the target ROI was drawn to cover the entire midsagittal plane of the CC. Further, exclusion ROIs were drawn axially just superior to the seed ROIs to prevent streamlines from erroneously projecting into the shafts of either the electrode or fiber implantations in the cortex. To further exclude spurious streamline deviating from the main tract, additional exclusion ROIs were placed sagittally (lateral to the implant regions) and coronally (both anterior to the implant regions and posterior to the projection area of CC). Up to one million streamlines were seeded for each tractogram, with at least one thousand selected. The tractography used constrained spherical deconvolution for fiber reconstruction and probabilistic tracking using the IFOD1 function with standard settings in MRtrix3 (Tournier et al., 2019). The mean (± SD) streamline length was calculated. In some cases, tractography did not cover the full vertical path into the gray matter at the fiber and/or electrode position. These pathway discrepancies, as well as the distance between the connecting tracts in the CC, were measured manually from the FA image. Tractography was excluded for one subject (rat27.1) due to a failed dMRI scan. The tract lengths and manual adjustments are detailed in Supplementary Table S3.

#### 2.4.5 Axon diameter estimation from dMRI

The preprocessed multi-shell dMRI data sets were normalized by the voxel-wise average of the nine b = 0 images. For each b-value, the powder average was computed by taking the arithmetic mean of the signals across the 30 diffusion-encoding directions. Given the strong diffusion weighting, that is, the high b-values, signal contributions from the extra-axonal space were assumed to be negligible (Veraart et al., 2020) and the signal was modeled as originating solely from the intra-axonal compartment. The Spherical Mean Technique-3 (SMT-3) axonal signal model (Andersson et al., 2022), based on the spherical average of cylinders (Andersson et al., 2020; Fan et al., 2020; Kaden et al., 2016; Kroenke et al., 2004), was fitted to the powder average signals using non-linear least squares. We were unable to fit the datasets for two subjects (rat33.1 and rat13.2).

Three parameters were fitted as part of the model: the intrinsic diffusivity, that is, intra-axonal axial diffusivity (*D_0_*), the intra-axonal perpendicular diffusivity ($D_{⊥}$), and the signal fraction of the intra-axonal space ($v_{a}$). The diameter could then be calculated from $D_{⊥}$. The intrinsic diffusivity was measured in individual brains from the principal direction within the CC region using the diffusion-tensor model fitted to the b-value of 4000 s/mm^2^, and was kept constant for the fitting of axon diameter (Alexander et al., 2010). Although it is inherently difficult to obtain an accurate estimate of the intra-axonal intrinsic (or axial) diffusivity, the estimated diameter, d, scales approximately as $d\propto D_{0}^{1/4}$ (Veraart et al., 2020), meaning that the diameter estimate is somewhat insensitive to the exact value of *D_0_*. We have previously shown this weak dependence of the diameter estimate on *D_0_* with simulations (Andersson et al., 2020).

Finally, we estimated the axon diameter from dMRI in the bilateral M1 projection region of CC. A 3D ROI, automatically mapped by the streamlines from tractography, was placed in the projection region of CC. Axon diameter estimates below 0.1 µm were discarded.

### 2.5 Histology

A subset of the brains (N = 7) was manually sectioned into two hemisphere slabs (3–4 mm thick).

#### 2.5.1 Fluorescent LM—locating the transcallosal fibers of M1

The right hemisphere (N = 3) was sectioned sagittally into 40 µm-thick slices. The fluorescence LM images were obtained on a fluorescence microscope (Nikon Eclipse 80i; Nikon Europe BV, Amsterdam, NL) with a 10x lens (NA: 0.30, Nikon Plan Fluor) through an FITC filter (536 nm; 515-558 nm) using the attached digital camera (1360 px × 1024 px; DP72; Olympus Europe, Hamburg, DE). Inspection of the sagittal slices revealed enhanced yellow fluorescent protein (EYFP) expression in the dorsal-anterior midbody of CC and informed the subsequent puncture procedure. A “super image” (N = 1) was obtained using a series of 10x magnification images that were stitched. In two animals (rat09.4 and rat10.1), the EYFP signal was too low compared to background, to obtain proper fluorescence microscopy images.

#### 2.5.2 Histological preparation for TEM

##### 2.5.2.1 Epon embedding of fixed tissue (“Epon-TEM”)

The midsagittal slab from the left hemisphere (N = 7) was punctured using a Ø = 1 mm biopsy puncture tool covering the region corresponding to projection locus of CC. The samples were embedded in 2% Agar, transferred to a 2.5% solution of glutaraldehyde in cacodylate buffer (0.1 M), and stored for 2 weeks. Thereafter, Epon embedding was conducted following a standard procedure (Riise & Pakkenberg, 2011). Sample preparation for two animals was unsuccessful (rat27.2 and rat13.2). Ultra-thin sections (~40–70 nm) used an ultramicrotome (Ultracut UCT or EM UC7, Leica Microsystems, Wetzlar, Germany). The sections were stained on the grids with uranyl acetate and lead citrate (EM AC20; Leica Microsystems, Wetzlar, Germany).

##### 2.5.2.2 Cryofixation of fresh tissue (“Cryo-TEM”)

Four additional young adult (~60 days old) rats (NTac:SD-M) were perfusion fixed for Cryo-fixation and embedding. Note, this cohort of rats did not undergo stereotaxic surgery or Opto-ElPhys experiments. The perfusion with 4% formaldehyde was carried out for only 3 min at 15 mL/min. The brain was extracted and placed in ice cold KPBS. Hereafter, we obtained 200 µm-thick slices of the midsagittal slab with a vibratome, and a similar region of the CC as for “Epon-TEM” was sampled using a Ø = 2 mm biopsy punch. Following approximately 2 h of storage in cooled KPBS, the samples underwent high-pressure freezing (HPM100; Leica Microsystems, Wetzlar, Germany), immediately followed by freeze substitution (AFS2; Leica Microsystems, Wetzlar, Germany) using 1% OsO_4_ in acetone, and consecutive Epon infiltration and embedding of the sample. Ultrathin sections (50–70 nm) were made with an ultra-microtome. Contrast enhancement was performed using a double staining protocol with uranyl acetate and lead citrate solutions (EM AC20; Leica Microsystems, Wetzlar, Germany).

##### 2.5.2.3 TEM of Epon- and Cryo-fixated samples

The “Epon-EM” and “Cryo-EM” images were obtained on a CM100 microscope (Philips; FEI, Eindhoven, NL) at 4200x magnification, captured in a sequential manner, to cover a large proportion or the entire CC cross-section in the Ø = 1 mm punctured sample at an image FOV of ~25 µm × 25 µm. We collected between 16 images (N = 1) to 69-77 images (N = 4), and 70–80 images (N = 4) per animal for Epon-TEM and Cryo-TEM, respectively.

##### 2.5.2.4 Segmentation, diameter and g-ratio estimation

The AxonDeepSeg (Zaimi et al., 2018) method was applied to the Epon-TEM images to segment axons and myelin. For the Cryo-TEM images, the AxonDeepSeg method did not work due to differences in tissue contrast. Instead, we trained a U-Net model on 15 images via an expert-in-the-loop procedure (Amirrashedi et al., 2025). Images were downsampled by a factor of four to be memory efficient. Initial ground truth annotations were derived from manually corrected predictions generated by AxonDeepSeg (Zaimi et al., 2018). The U-Net was configured with a kernel size of 5 × 5 pixels and four output classes: unlabeled background, labeled background, axon, and myelin. The training was performed using 30 patches per image, each of size 384 × 384 pixels, randomly sampled with a stride of 20 × 20 pixels. The loss function is a modified, masked Generalized Dice Loss based on MONAI’s (Cardoso et al., 2022) implementation. The U-Net model implementation used PyTorch (Ansel et al., 2024), and TorchIO (Pérez-García et al., 2021) facilitated efficient data handling.

Post-processing was applied to the segmentation results to include only fully segmented and myelinated axons. First, a blob analysis isolated individual myelinated axons and separated the myelin of adjacent fibers. Axons lacking myelin and myelin regions without axons were excluded. Axons overlapping the 4-pixel border region of the image were excluded. Standard morphological and intensity operations were employed to remove holes in segmented axons. Axons were rejected if myelin surrounded less than 95% / 85% of the perimeter or if the eccentricity was larger than 0.92 / 0.975, for Epon-TEM and Cryo-TEM respectively. Finally, inner diameter, myelin thickness, and the g-ratio were estimated from the minor axis of an ellipse fitted to each blob, accounting for shape eccentricity.

### 2.6 Calculating the structure-function relationship between conduction velocity and axon diameter

From the obtained length of the projection pathway (L) and the measured TCT, we calculated the estimated conduction velocity (CV) as follows:

CV =L/​TCT (1)

Similarly, we calculated the CV along the pathways using the measured g-ratio and axon diameter, given the structure-function relationship by Waxman and Bennett (1972):

CV=(5.5​/​g) · d (2)

where g, that is, the g-ratio, was set to a group mean of TEM data results (g = 0.64), rather than the literature value (Andersson et al., 2020; Caminiti et al., 2013; Tettoni et al., 1996) of ~0.7. Hence, combining Eq. (1) and (2) allows us to predict the TCT from structural measures, or the converse prediction of axon diameter from the functional measure.

### 2.7 Axon diameter distributions from histology

We applied a kernel density estimation (KDE) on the distribution data to obtain the distribution mode. Statistical features were thus expressed as the mean, SD, and the mode. We additionally refer to the larger axons from the distribution by the 90^th^ percentile (p90). Shrinkage was calculated for both the mode and the p90 group means: (CryoTEM-EponTEM)*100/CryoTEM. The general shrinkage factor between Epon-TEM and Cryo-TEM was calculated as the average of these two shrinkages (mode and p90). The voxel-wise dMRI diameter estimates are weighted toward larger diameters (Alexander et al., 2010; Burcaw et al., 2015; Lee et al., 2020; Packer & Rees, 1972; Veraart et al., 2020). We accounted for this weighting by calculating the weighted diameter (d_w_) from the histological axon diameter distributions. In the wide pulse limit, δ≫R^2^⁄D_0_ (Burcaw et al., 2015; Lee et al., 2020; Veraart et al., 2020) where R is the axon radius from histology, D_0_ is the intrinsic diffusivity and δ is the pulse-width of the diffusion encoding. In practice, this wide-pulse limit applies to most axons in the rat brain (Andersson et al., 2022). Using this assumption, we can calculate d_w_ as (Burcaw et al., 2015; Veraart et al., 2020):

$$d_{w}=2\;{\left(\frac{<R^{6}>}{<R^{2}>}\right)}^{0.25}\;$$ (3)

### 2.8 Simulation of sensitivity profile of dMRI to diameters

To assess the lower bound of measurable axon diameter, we calculated the sensitivity profile to axon diameter of this dMRI acquisition as previously described in detail (Andersson et al., 2022). Briefly, the signals from cylinders were analytically generated for diameters in the range 0.2–15.0 μm at 0.2 μm steps. Rician noise of a given signal-to-noise ratio (SNR) was added to the signals, and the powder average cylinder model was fitted to obtain a diameter estimate, termed SMT-3 in Andersson et al. (2022). The Rician distributed noise was emulated by calculating the magnitude of complex Gaussian distributed noise in which the standard deviations of the real and imaginary components were both 1/SNR. The diameter estimation was performed 50 times for each diameter, with each repeat represented by a green data point in Figure 3h. The sensitivity profile revealed a diameter lower bound of around 2 μm and an upper bound of around 6 μm for accurate estimation of the diameter.

## 3 Results

### 3.1 Recording the transcallosal conduction time

**Fig. 1.**
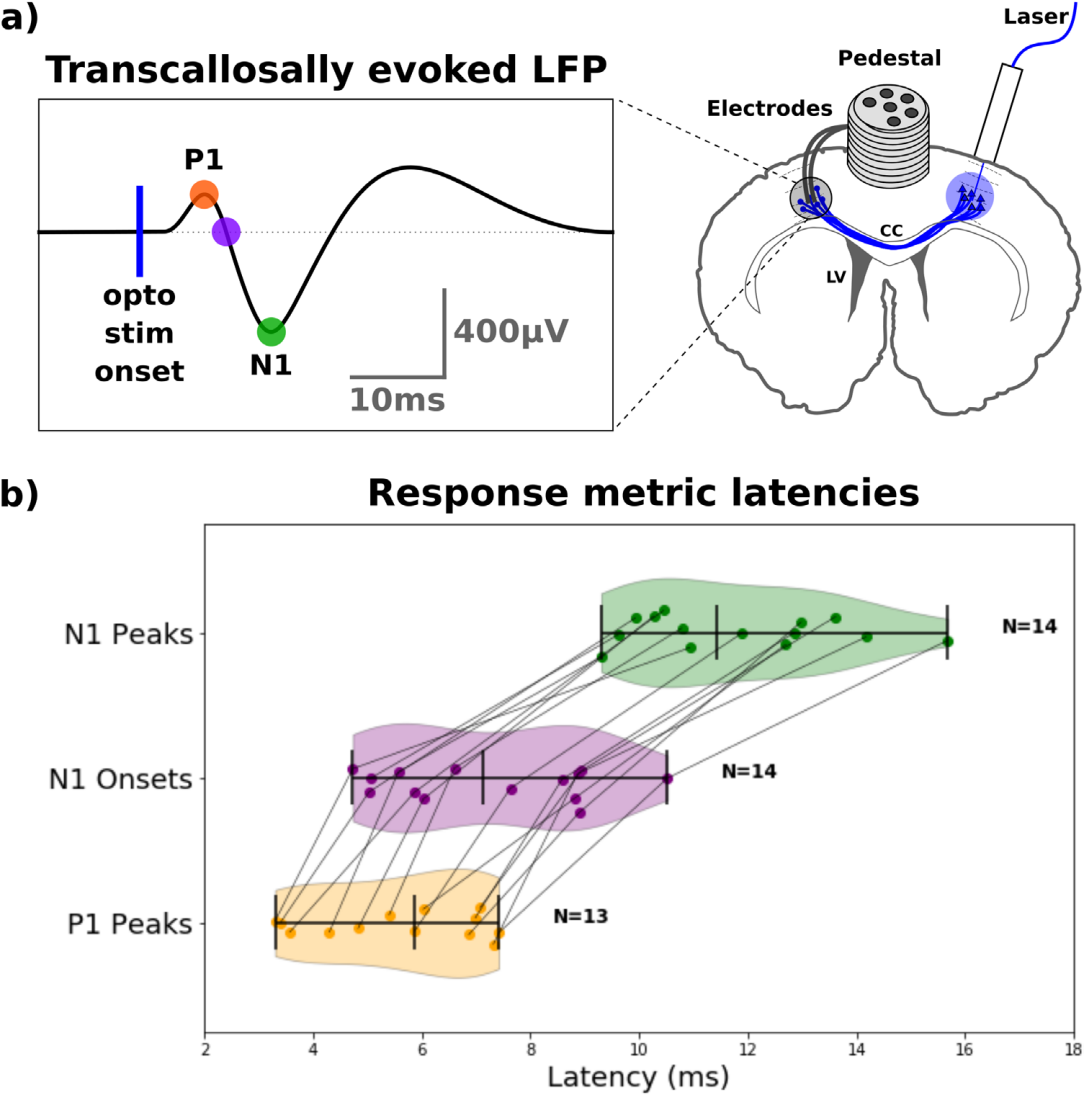
Conduction latencies obtained through optogenetic stimulation and electrophysiology, measured as local field potentials (LFP). (a) Optogenetic stimulation of the right M1 and electrophysiological recording in the left M1. Orange dot: P1 peak; Purple dot: N1 onset; Green dot: N1 peak. (b) Plot of median values of peak detections for all animals (Skoven et al., 2022) in reliable conditions, with a coefficient of variation for the N1 peak being <1.00. Colors correspond to the insert in subfigure (a). The group means are depicted as the middle vertical line in each of the three metrics, and the outer-most vertical lines represent the minimum and maximum values for each metric.

The transcallosal evoked responses were recorded in 16 animals, as outlined in Figure 1a. The latencies (i.e., the P1 peak, N1 onset, and N1 peak) were obtained (Supplementary Table S1) from stimulation conditions that produced a reliable N1 peak. This typically included higher stimulation intensity (≥1 mW) and/or longer stimulation durations (≥1 ms). The mean latency ± mean of *within*-animal SD was 5.6 ± 0.3 ms for P1 (N = 13), 7.2 ± 0.3 ms for the N1 onset (N = 14) and 11.8 ± 0.5 ms for the N1 peak (N = 14), respectively, as shown in Figure 1b. The P1 peak was the earliest transcallosal response that was consistently detectable across most animals (N = 13) and was thus used as an indicator of the transcallosal conduction time (TCT). In comparison to the *within*-animal SD of the latencies, the *between*-animal SDs were relatively large, being 1.49, 1.81, and 1.84 ms for the P1 peak, N1 onset, and N1 peak, respectively. Hence, higher variability was between animals than within. As the variation between animals might reflect differing electrode depths, we measured the electrode depths on high-resolution structural T2-weighted MR images. The mean (± SD) depth was found to be 1045 ± 48 µm (N = 5; Supplementary Table S2).

### 3.2 Mapping the pathway length to estimate the conduction velocity

**Fig. 2.**
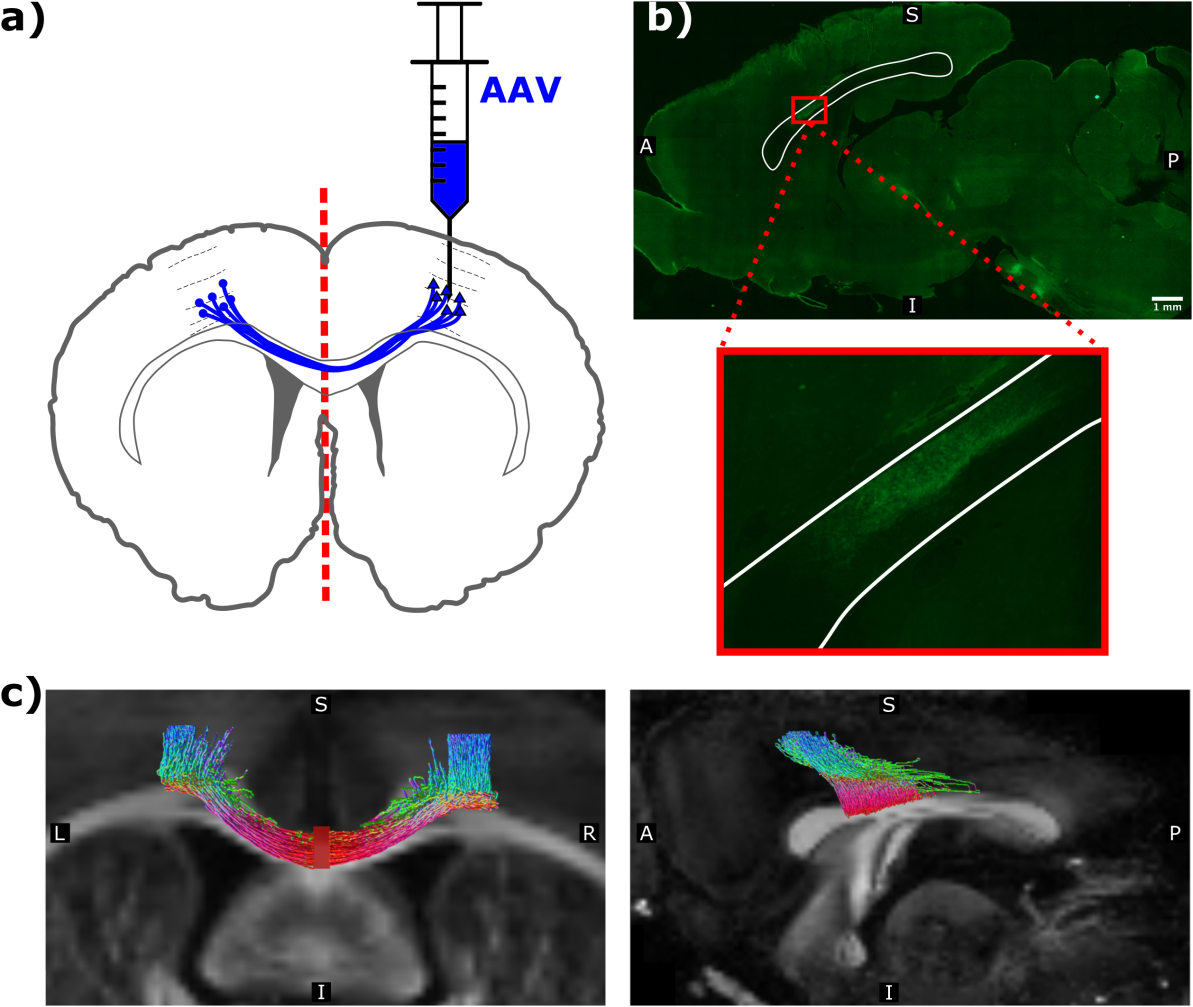
Viral expression and mapped projection pathway of the corpus callosum. (a) Illustration of the viral injection in the right M1. (b) Digitally stitched mid-sagittal image composed of multiple 10x magnification images acquired by fluorescence microscopy traversing the whole slice of rat14.5. Green corresponds to the expression of enhanced yellow fluorescent protein (EYFP) from the virally transfected neurons, highlighting the projection pathway between the primary motor cortices through the CC. The insert figure is an enlarged view of the small red box in the top panel. White lines delineate the contour of the mid-sagittal CC. Scale bar corresponds to 1 mm. (c) Coronal (left panel) and sagittal (right panel) view of the dMRI scan of rat33.1, overlaid with the tractography streamlines projecting between the seeding region (located in the left M1 and right M1), projecting toward and terminating in the mid-sagittal region (filled red rectangle in the coronal section, left). The color-coding of tractography streamlines indicates their local direction, with red being left/right, green being anterior/posterior, and blue being superior/inferior.

The viral injection in the right primary motor cortex (M1) targeted excitatory neurons (through the CaMKIIα promoter) projecting through the CC to the contralateral M1 (Fig. 2a). Since these optogenetically stimulated neurons (Fig. 1) were fluorescence-labeled (Fig. 2b), their trajectories could be detected through the mid-body of the CC and was present in the superior half of the anterior midbody of CC (Fig. 2b, magnified in the red box insert).

Figure 2c shows that tractography was able to reconstruct the interhemispheric connection between the stimulation (right M1) and the recording sites (left M1). Indeed, a visual inspection of Figure 2c confirmed that the tractography overlapped with the CC projection region from fluorescence-labeled axons. The tractography-derived connection length across all animals was 11.47 ± 0.47 mm (mean ± SEM; N = 8; Supplementary Table S3).

### 3.3 Measuring the axon diameters and g-ratios

Conduction velocities were predicted from structural measurements, using Eq. (2). In a sub-group of the animals (N = 4), axon diameter estimates of the same brain were obtained from both *post mortem* dMRI and subsequently axon diameter and g-ratio measures from Epon-TEM images. Some animals were exposed to only TEM (N = 1) or only dMRI (N = 3).

**Fig. 3.**
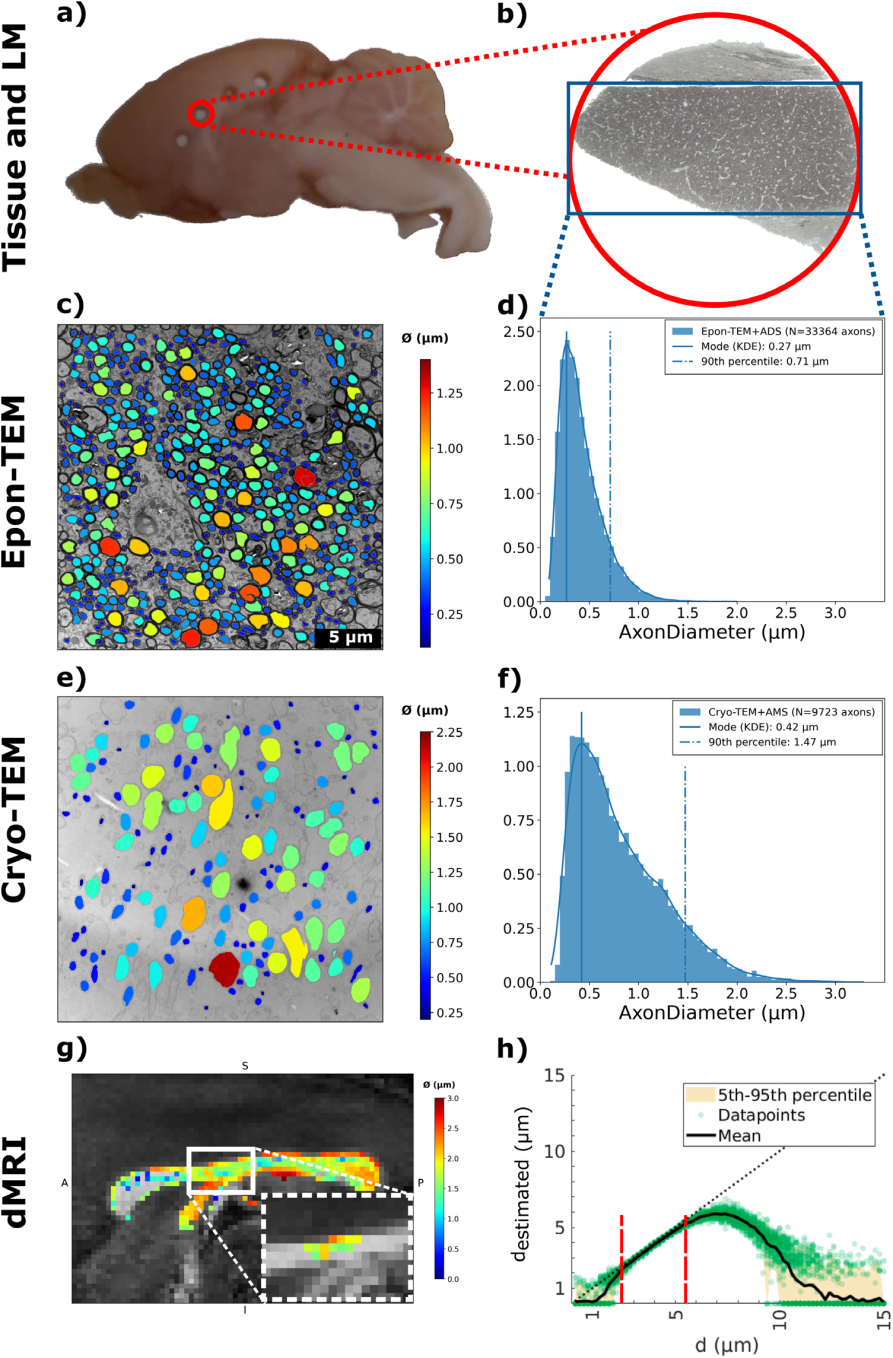
Axon diameter measurements in corpus callosum using different imaging techniques. (a) Mid-sagittal slab (~3 mm thick) with Ø = 1 mm punctures. (b) Cross-section of the EPON-embedded puncture, imaged with light microscopy at 10x magnification. (c) Epon-TEM image acquired at 4200x magnification from rat27.3. Segmentation of axons is colored according to their diameter (color bar to the right). (d) Histogram of the distribution of axon diameters in CC automatically segmented and estimated with AxonDeepSeg of all TEM images of this animal. (e) Overview TEM image from Cryo-fixated and -embedded CC-tissue (Cryo-TEM) from a different animal (rat1.4). (f) Axon diameter distribution from all Cryo-TEM images from this animal. (g) dMRI axon diameter distribution from rat14.1 of whole CC. A = anterior, P = posterior, S = superior, I = inferior. The white box insert indicates the ROI voxels determined by tractography from the same rat. (h) The sensitivity profile to diameter of the dMRI acquisition for Rician noise with a signal-to-noise ratio of 50, showing the estimated cylinder diameter (d_estimated_) vs. the ground truth diameter, d. The red dashed lines indicate approximate lower and upper bounds outside of which diameter estimation shows high uncertainty.

Figure 3c and 3d show the axon segmentation results of one of 69 Epon-TEM images from rat27.3 and the corresponding histogram of the axon diameter distribution from all images (N = 33364 axons) of that animal. Though AxonDeepSeg detects a vast number of axons, not all axons in each image were detected. The KDE applied to the distribution revealed the mode of the axon diameter distribution (the peak of the KDE) of all TEM images being 0.27 µm. The large axons, that is, the tail of the histogram represented by the 90% percentile is 0.71 µm. Minimal difference is observed between animals having a group mean (± SD) of the modes obtained from the KDEs 0.31 ± 0.04 µm and a 90% percentile of 0.66 ± 0.05 µm (N = 5; Supplementary Fig. S2).

To explore the tissue shrinkage factor in conventional TEM due to sample preparation (especially dehydration), we compared the results with cryo-fixation that better preserve the *in vivo*-like state of the tissue.

Figure 3e and 3f show the axon segmentation results in one Cryo-TEM image of 84 from rat01.4, and its corresponding histogram distribution for all images (N = 9723 axons). The mode obtained from the applied KDE was found to be 0.42 µm. Between animals, the group mean (± SD) of the mode and the 90% percentile are 0.39 ± 0.03 µm and 1.42 ± 0.18 µm respectively (N = 4; Supplementary Fig. S3).

We find a shrinkage factor between Epon-TEM and Cryo-TEM of 21% for the mode and 53% for the 90% percentile, suggesting an axon diameter dependent shrinkage where larger axons shrink more. We applied a general tissue shrinkage factor calculated as the average of the two being 37%.

The g-ratios were estimated from the TEM segmented axons (Supplementary Fig. S1), which produced a group mean (± SD) of the individual g-ratio distribution means of 0.64 ± 0.04 (N = 5). Axon diameter distributions as well as g-ratio distributions overlapped between animals (Supplementary Figs. S1-S3).

Using dMRI we estimated the diameter along the bilateral M1 projection within individual trajectory ROIs, delineated by tractography between the stimulating and recording regions (Fig. 2c). Figure 3g shows axon diameter estimates in a midsagittal slice of the CC, and an outline of the tractography ROI where M1 projects through. Across the CC, as seen in Figure 3g, as well as along the contralateral M1 tract, some voxels show axon diameters of zero. Excluding these voxels along the M1 projection, the distribution mode of this animal (rat14.1) was 1.34 ± 0.42 µm (mode ± SD). The voxel distributions of all animals are shown in Supplementary Figure S4. Figure 3h shows the simulated expected sensitivity profile of the experimental dMRI setup to axon diameter when accounting for the acquisition parameters and SNR (Andersson et al., 2022). The lower and upper diameter bounds of the sensitivity range were around 2 and 5.5 µm, respectively. Below the lower bound, there was a broader transition area between 1–2 µm with large uncertainty in diameter estimations (Fig. 3h). Hence, MRI voxels in which the diameter is estimated to be zero could potentially correspond to diameters of 2 µm or lower.

### 3.4 Multi-modal structure-function relationship

**Fig. 4.**
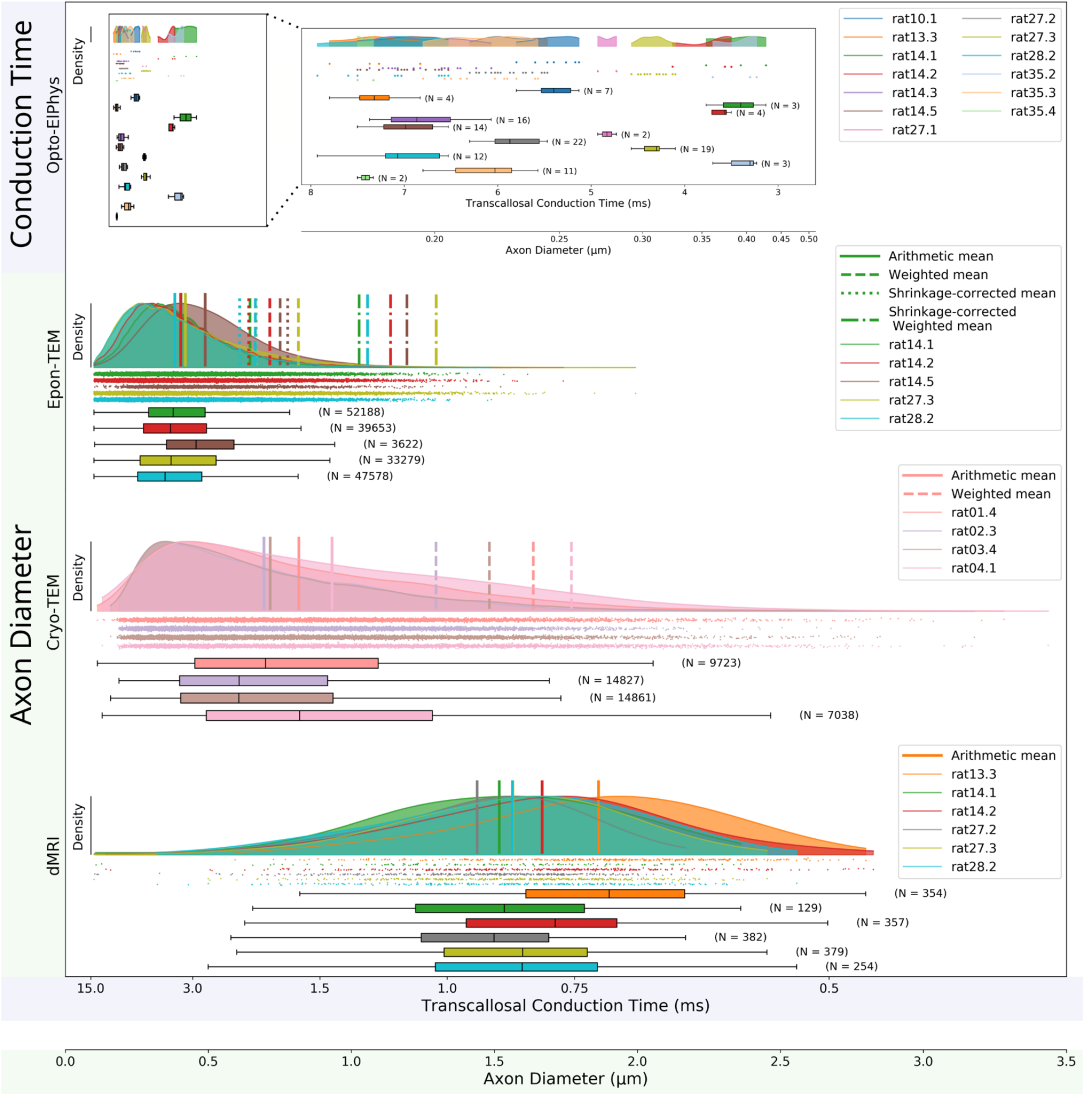
Structure–function relationships of measured and predicted values across all modalities applied to the rat brain. In the main figure, data points (“N”; dots below the kernel density estimations) represent either predicted (for Opto-ElPhys) or measured axon diameters (for TEM and dMRI) corresponding to the second x-axis (highlighted by transparent green). The first x-axis (highlighted by transparent blue) corresponds to predicted Transcallosal Conduction Time (TCT) computed using the fixed g-ratio (g = 0.64; N = 5 rats) and the mean pathway length from dMRI tractography (L = 11.47 ± 0.47 mm; N = 8 rats). Each distribution corresponds to one animal (color-coded). In the Opto-ElPhys row, diameters are predicted from measured TCTs, individual pathway lengths, and the fixed g-ratio. In the Opto-ElPhys insert, the plotted values correspond to the measured TCT values, with the second x-axis showing predicted diameters based on the same parameters. For axon diameter distributions in Epon-TEM, Cryo-TEM, and dMRI, the arithmetic means are marked with solid lines. For Epon-TEM and Cryo-TEM, the weighted means (for comparison to dMRI) are dashed. For Epon-TEM, the shrinkage-corrected means and shrinkage-corrected weighted means are shown as dotted and dash-dotted lines, respectively. Note: TEM and dMRI capture fundamentally different quantities—TEM shows actual axon diameter distributions in tissue, while dMRI reflects voxel-wise diameter indices across the selected ROI.

We map the structure-function correlation between the axon diameter distributions from the different structural image modalities (dMRI, Epon-TEM, Cryo-TEM) and functional measure (TCT), as well as their corresponding predicted counterparts as shown in the x-axes of Figure 4. The conversion between the measured and the predicted TCTs and axon diameters uses Eq. 1 and Eq. 2 in the Section 2 and uses the group mean g-ratio (0.64 ± 0.04; N = 5) obtained from the Epon-TEM results and the group mean pathway length (11.47 mm ± 0.47, N = 8) from tractography.

The prediction of the mean axon diameter from the group means (± SD) of TCTs (5.57 ± 1.49 ms; N = 13) corresponded to axons with mean (± SD) diameters of 0.26 ± 0.08 µm (N = 13)—with predicted axon diameters ranging from 0.17 µm to 0.46 µm. Such diameters, thus, extend from below and up to the mode of the diameter distributions quantified with TEM (row 1 vs. rows 2 and 3).

For shrinkage correction of axon diameters obtained from Epon-TEM, we applied the measured general shrinkage factor of 37% to the axon diameter distributions (N = 5). Shrinkage-corrected means of the Epon-TEM axon distributions (vertical dotted lines, Fig. 4, row 2) are shifted closer and, thus, show good correspondence to the arithmetic means of Cryo-TEM (Fig. 4, row 3; vertical solid lines, Supplementary Fig. S3).

The arithmetic mean voxel distribution of dMRI-estimated diameters shown in Figure 4 (row 4) have no simple relation to the predicted TCT, due to the volume-weighting of the dMRI diameter estimate toward larger diameters related to Eq. 3 (Methods). The weighted mean of the axon diameters from shrinkage-corrected Epon-TEM (dash-dotted vertical lines, Fig. 4, row 2) and weighted mean from Cryo-TEM (dashed vertical lines, Fig. 4, row 3) generally agree, but are lower than those obtained from dMRI. Note, the smaller diameter axons, likely responsible for the measured TCT in Figure 4 (row 1), are not directly quantifiable with dMRI due its sensitivity profile excluding the smallest diameters shown in Figure 3h.

The between-animal variation of the dMRI-estimated diameters is larger than the TEM results obtained on the same animals. This suggests that axon diameters detected by dMRI in some animals mostly fall within the transition region of the sensitivity profile (Fig. 3h), which likely accounts for the large between-animal variation.

## 4 Discussion

Focusing on motor transcallosal fibers, we found that conduction time quantified from LFPs in the same rat brain structurally correspond to a subpopulation of smaller axons. Specifically, those with diameters below or up to the mode of the distribution that we measured with TEM—when converted using the structure-function relationship (Eq. 2) by Waxman and Bennet (1972). Likewise, although we demonstrate the presence of larger axons using both EM and dMRI, that is, the tail of the axon diameter distribution, the predicted short TCTs were not reflected in the measured LFP-based TCTs. Importantly, performing electrophysiology (TCT measurement), dMRI (axon diameter estimation), and TEM with classical Epon embedding (axon diameter and g-ratio measurement) on the same animals allows us to attribute the large variations in the respective estimated axon diameter distributions to differences in sensitivity between modalities, rather than anatomical differences. Cryo-TEM of tissue from additional rats revealed that the degree of axonal shrinkage in regular Epon-TEM is diameter-dependent. Altogether, our findings highlight that the sensitivity profiles to structure (diameter) and function (latencies) of each measurement modality may differ when predicting one from the other. Recognizing and accounting for these modality-dependent sensitivity differences enhances the precision with which structural and functional changes are linked, allowing the detection of subtle, previously unresolved variations in the structure–function relationship. This integrative perspective provides a more nuanced view of how structural and functional measurements relate, setting the stage for future work to refine the models and bridge remaining gaps between microstructural features and neural function.

### 4.1 Predicting axon diameters from the transcallosal conduction times

We used the peak of the first positive deflection of the transcallosal response, P1, as our measure of TCT. Within animals, P1 latencies were stable on a sub-millisecond scale (SD: ± 0.26 ms), despite use of different stimulation parameters (Skoven et al., 2022). Between animals, however, TCTs varied by several milliseconds (SD: ± 1.49)—a variation not fully explained by the minor pathway length differences from tractography (Caminiti et al., 2013; Pesaresi et al., 2015; Tang-Wright et al., 2022).

To record the evoked LFP response, we used stereotrodes with a fixed implantation position. High-resolution *ex vivo* MRI confirmed a small electrode depth variation (SD <50 µm, i.e., ~5%, of the intended depth of 1 mm in M1) relative to the resolution of the digital stereotaxic surgery frame (10 µm). However, we cannot rule out that even such minor depth differences contributed to the observed TCT variation between animals (Iordanova et al., 2018). Despite inter-animal variability in TCT, our results align with previous studies using electrical stimulations: Hoffmeyer et al. (2007) reported sensory transcallosal LFP latencies of 4–11 ms using superficial electrodes, while Seggie and Berry (1972) found ~8 ms latencies with surface cortical recordings.

Interestingly, TCTs measured in humans using TMS, as well as those measured (Swadlow et al., 1978) or predicted (Caminiti et al., 2013; Tomasi et al., 2012) in monkeys, are comparable to those in our rat study. This cross-species similarity suggests structural or functional adaptations, as interhemispheric distances increase with brain size. While the largest CC axons scale with brain size (Caminiti et al., 2009; Olivares et al., 2001; Perge et al., 2012), the modal axon diameter remains relatively constant across mammals (Barazany et al., 2009; Caminiti et al., 2009; McDougall et al., 2018), and even tissue shrinkage (37%) cannot fully explain the conserved TCTs despite the ~750-fold difference in brain volume between humans and rodents (Dyrby et al., 2011, 2014, 2018). Applying Eq. (1) and (2) to human TCTs (5–13 ms; (Di Lazzaro et al., 1999; Ferbert et al., 1992; Rogasch et al., 2014)) and interhemispheric pathway lengths (111.4–122.4 mm; (Caminiti et al., 2009, 2013)) predicts axon diameters of 1.09–3.12 µm—above the modal value and consistent with preferential activation of large excitatory neurons by TMS (Peterchev et al., 2013; Siebner et al., 2022). In contrast, our rat LFP-based recordings link TCTs to axons below the mode, implying that conserved TCTs cannot be explained by simple scaling of axon diameter. Rather, they may reflect contributions from distinct axon populations with different latencies (Andersson et al., 2020), highlighting the need to consider both the specificity of stimulation/recording methods and the full axon diameter distribution when inferring conduction properties.

### 4.2 Measured axon diameter distributions depend on the tissue preparation

Using TEM, we observed consistent axon diameter distributions across rats in the midbody corpus callosum, in line with previous studies using conventional tissue preparation (see Supplementary Table S4). Olivares et al. (2001) reported similar modal diameters (0.11–0.2 µm) across species from rats to cows. In contrast, larger modal diameters (~1 µm) have been reported in the rat midbody using EM (Barazany et al., 2009) and in the genu using confocal microscopy of hydrated tissue sections (Veraart et al., 2020). These values exceed even our Cryo-TEM estimates, suggesting that tissue processing, imaging resolution and field-of-view (Kjer et al., 2025), as well as regional anatomical variation (Dyrby et al., 2014, 2025), all influence the measured distributions.

We observed non-uniform shrinkage across the axon diameter distribution, with larger shrinkage in the tail (90th percentile) than near the mode. This may reflect greater water content in large axons, making them more vulnerable to dehydration during Epon-TEM processing. While shrinkage estimates in white matter vary widely (0–65%; (Caminiti et al., 2009; Dyrby et al., 2018; Houzel et al., 1994; Tomasi et al., 2012)), they are typically assumed to be diameter-independent. A full characterization of the diameter-dependent axonal shrinkage is essential for translating histology- or TEM-derived axon diameter distributions to the *in vivo* state. To account for this effect, we applied an average correction factor of 37% (Supplementary Fig. S2), based on the relative shrinkage of the distribution mode and its 90th percentile. This yielded good agreement with our Cryo-TEM data in the rat, where the tail of the axon diameter distribution is less pronounced. It is likely less appropriate for other species, for example, primates, where the prevalence of large axons is higher (Caminiti et al., 2009, 2013).

Notably, *ex vivo* dMRI is performed on fixed, hydrated tissue assumed to be close to the *in vivo* condition, which is why the estimates are typically corrected for shrinkage when compared with EM (Alexander et al., 2010; Dyrby et al., 2018). However, it is known that keeping tissue in fixative (formaldehyde) over time can lead to over-fixation, introducing the risk of tissue shrinkage. Our fixed tissue, being MRI-scanned, has been stored for a limited time in cold PBS to reduce over-fixation, and we do not observe clear tissue shrinkage.

### 4.3 The structure–function relationship with dMRI

dMRI-derived axon diameter estimates were consistently larger than those obtained from electrophysiology or TEM, with distribution modes between 1.3 and 2.2 µm (Fig. 4, bottom row). These values predict conduction latencies ≤1 ms (Eq. 2), faster than what could be measured from LFPs with our setup. The two modalities also provide inherently different diameter/latency estimates: dMRI provides one weighted mean over axon diameter distribution per image voxel (Alexander et al., 2010; Burcaw et al., 2015; Lee et al., 2020; Packer & Rees, 1972; Veraart et al., 2020). In contrast, our electrophysiology setup captures the integrated response from a large axonal population, with the synchronized activity from axons at the mode of the diameter distribution likely being the major contributor to the peak. For the TEM distributions, the MR-weighting (Eq. 3) could be taken into account. This yielded better agreement between dMRI and Cryo-TEM estimates than with shrinkage-corrected Epon-TEM—likely due to residual errors in the applied average shrinkage correction. Our prior work has shown that 2D diameter measurements correspond well with the 3D mean axon diameters (Andersson et al., 2020), supporting the comparison of 2D TEM with 3D dMRI-based estimates. However, few Epon-TEM axons exceeded the dMRI sensitivity threshold (~2 µm), even though we imaged a broad FOV (10000–47000 µm²) with Epon-TEM. This covered almost the entire CC within the 1 mm biopsy punch, to sample the full diameter range and prevent methodological bias toward the more densely packed smaller axons (Andersson et al., 2020; Lamantia & Rakic, 1990; Veraart et al., 2020) (Supplementary Fig. S5).

Further limiting the comparability of dMRI-based axon diameters with the other modalities is its selective sensitivity to axons above a lower bound determined by gradient strength, acquisition parameters, SNR, and modeling assumptions (Andersson et al., 2022; Dyrby et al., 2013; Fan et al., 2020; Nilsson et al., 2017; Sepehrband et al., 2016). Simulations (Fig. 3h) show a “transition area”, below ~2 µm, where estimates are frequently zero or uncertain. In our data, some voxels returned zero estimates and there was large variability within each ROI (Fig. 4, bottom row), suggesting a dominant population of small, sub-threshold axons. The lower bound can be reduced with stronger gradients; for example, Veraart et al. (2020) achieved ~1.6 µm using 1500 mT/m, reporting a mean of ~2.5 µm in the rat CC, consistent with the present study. In humans, dMRI-based mapping has shown high reproducibility (Fan et al., 2021), despite the fact that sensitivity remains limited to axons several micrometers in diameter (Alexander et al., 2010; Andersson et al., 2022; Fan et al., 2020; Pizzolato et al., 2023; Veraart et al., 2020). This is likely due to a higher prevalence of large-diameter axons in the human brain (Caminiti et al., 2009; Olivares et al., 2001), facilitating successful whole-brain mapping and alignment with functional estimates from TMS (Fan et al., 2020; Veraart et al., 2020).

### 4.4 Considerations

Several methodological factors should be considered when interpreting the electrophysiology results. Firstly, the optogenetic approach allowed for the selective stimulation of excitatory callosal neurons, in contrast to electrical stimulation which activates all neurons (Aravanis et al., 2007). However, although we confirmed strong EYFP expression in the CC projection area (Skoven et al., 2022), indicating high prevalence of the target neurons, off-target transfection remains possible due to the presence of surrounding pyramidal cells and inhibitory interneurons at the viral injection site (Barth et al., 2016; Jiang et al., 2015). Secondly, while optogenetics avoids stimulation artifacts and enables cell-type specificity (Aravanis et al., 2007), it introduces an activation delay (t_kON_) of 0.6–2.6 ms (Kleinlogel et al., 2011; Lin et al., 2009; Nagel et al., 2005) that depends on stimulation intensity. Because we used variable laser intensities and did not precisely measure onset delays, individual correction was not feasible. Although using fixed stimulation intensity might have reduced variability in the observed TCTs, the observed sub-millisecond within-animal differences suggest minimal impact. Nonetheless, accounting for onset delays would effectively shorten the measured TCTs, potentially providing better correspondence with the somewhat larger (and faster) axons detected by TEM. Optogenetic onset delay should be considered in future analyses, for example, by using optrodes to measure onset neuronal firing. Thirdly, the contribution of stimulated neurons to the TEM-segmented axons is unknown, but the low density of inhibitory neurons projecting through the CC (Rock et al., 2018) suggests limited impact on the LFP or diameter distribution.

In addition to the sensitivity profile of the dMRI acquisition (Fig. 3h) which complicates the diameter estimation of small axons, we also did not model the presence of the dot compartment, which has been shown to be present in *ex vivo* tissue (Alexander et al., 2010; Tax et al., 2020; Veraart et al., 2020).

Although the high b-values used here would have suppressed the signal from the extra-cellular compartment, the signal from the dot compartment (if any) would persist. Not accounting for it results in an artificial reduction of dMRI-estimated diameter but does not alter our conclusion that it remains sensitive to the tail of the axon diameter distribution. It is possible to be insensitive to the dot compartment at high b-values using a spherical harmonic model approach and zonal-ratios (Pizzolato et al., 2023). This approach would require at least two high b-values but more gradient directions than those used here, and has shown to be highly sensitive to noise.

## Supplementary Material

Supplementary Material

## Data Availability

Code is available at referenced repositories (https://gitlab.com/cskoven/PulsePal, https://gitlab.com/cskoven/opto-elphys, https://github.com/MaP-science/DeepAxonMyelinEMSegmenter/tree/skoven-c-2025). Data are publicly available in the Zenodo-data repository (https://doi.org/10.5281/zenodo.17476529).
