# Supplementary material for: Axonal structure-function relationships across experimental modalities
Source: Imaging Neurosci (Camb). 2025 Dec 22;3:IMAG.a.1058. doi: 10.1162/IMAG.a.1058 (PMC12723409; doi:10.1162/IMAG.a.1058)
Supplement: Supplementary Material [file IMAG.a.1058_supp.pdf]

Supplementary Information for
Axonal Structure-Function relationships across experimental modalities

Christian S. Skoven (1,2), Mariam Andersson (1), Miren Lur Barquin Torre (1), Marco Pizzolato (3,1), Hartwig R. Siebner (1,4,5) and Tim B. Dyrby (1,3)

(1) Danish Research Centre for Magnetic Resonance,
Department for Radiology and Nuclear Medicine,
Copenhagen University Hospital Amager and Hvidovre, Copenhagen, Denmark.

(2) Center of Functionally Integrative Neuroscience,
Department of Clinical Medicine, Aarhus University, Aarhus, Denmark.

(3) Department of Applied Mathematics and Computer Science,
Technical University of Denmark, Denmark.

(4) Department of Neurology, Copenhagen University Hospital Bispebjerg and Frederiksberg,
Copenhagen, Denmark.

(5) Department of Clinical Medicine, Faculty of Medical and Health Sciences,
University of Copenhagen, Denmark.

Correspondence: Christian S. Skoven (cskoven@drcmr.dk) and Tim B. Dyrby (timd@drcmr.dk)

This PDF file includes:

Figures S1 to S5
Tables S1 to S5
Supplementary Information References

Supplementary Figures

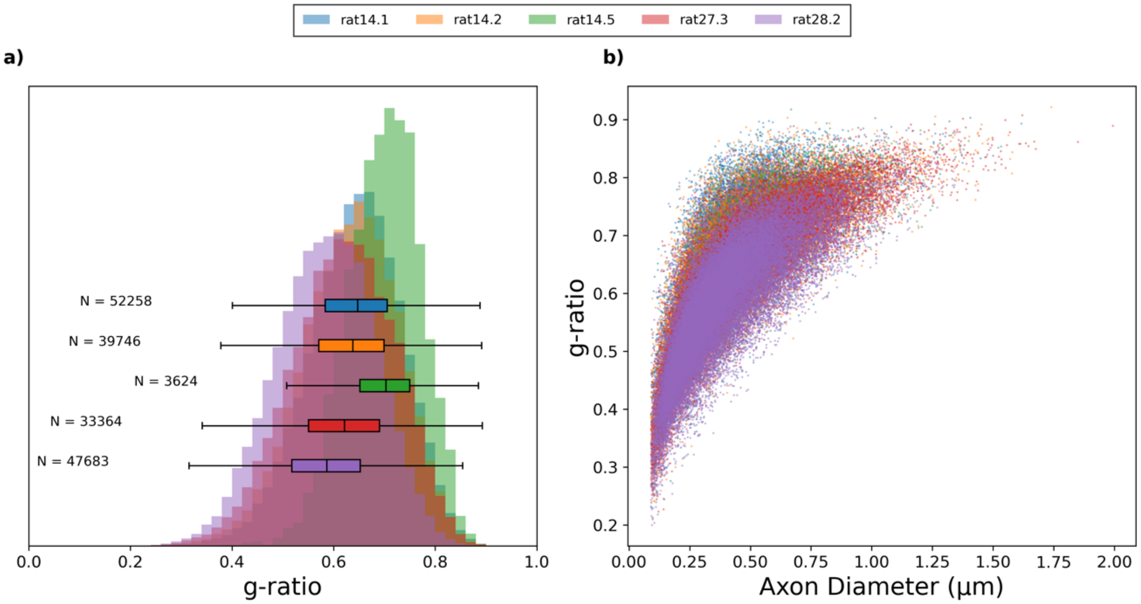

Fig. S1.: Axonal g-ratios, segmented from Epon-TEM. Distribution of g-ratios of the automatically segmented axons (Left panel). G-ratio vs axon diameter (Right panel).

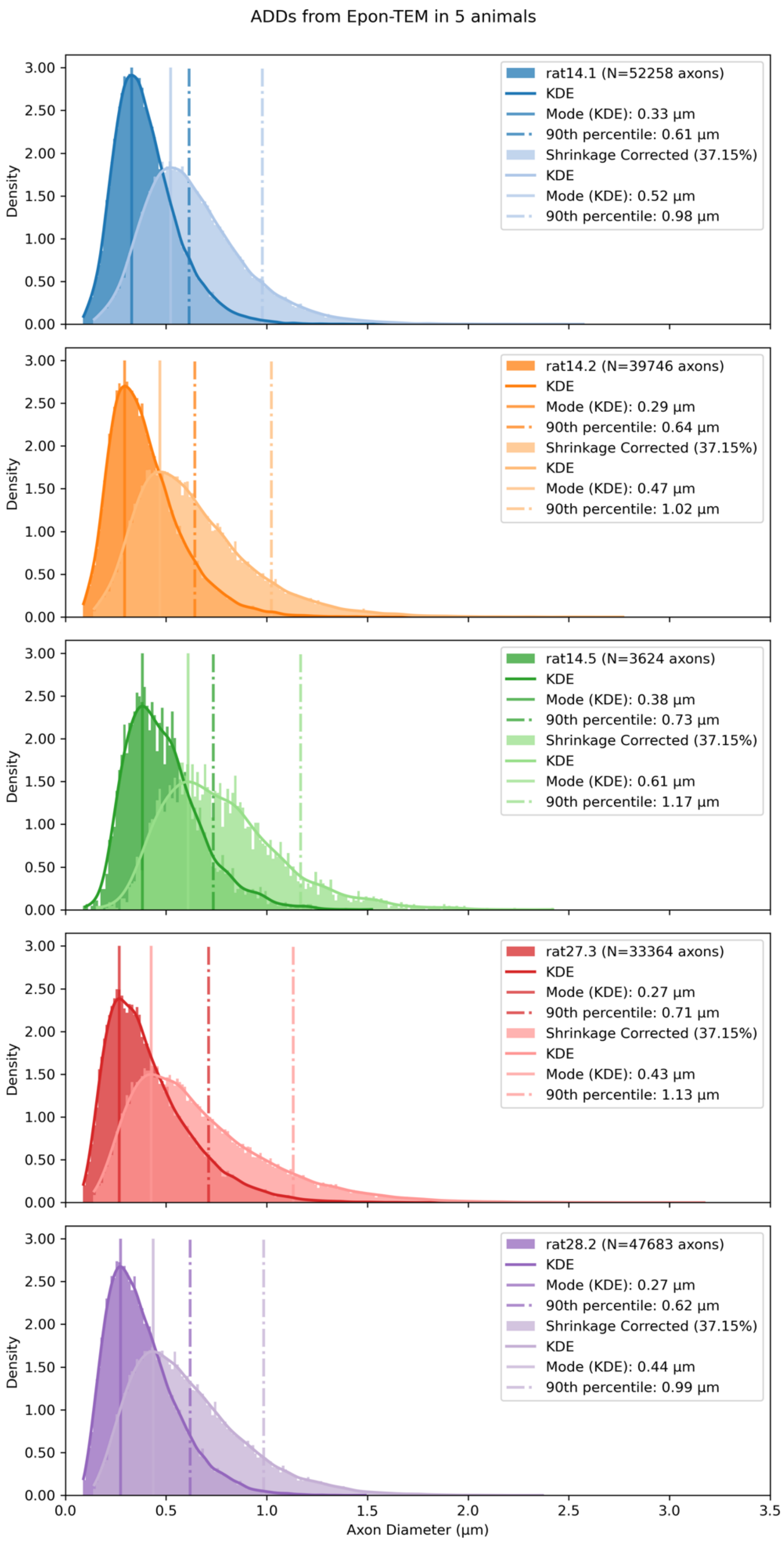
ADDs from Epon-TEM in 5 animals
rat14.1 (N=52258 axons)
KDE
Mode (KDE): 0.33 µm
90th percentile: 0.61 µm
Shrinkage Corrected (37.15%)
KDE
Mode (KDE): 0.52 µm
90th percentile: 0.98 µm
rat14.2 (N=39746 axons)
KDE
Mode (KDE): 0.29 µm
90th percentile: 0.64 µm
Shrinkage Corrected (37.15%)
KDE
Mode (KDE): 0.47 µm
90th percentile: 1.02 µm
rat14.5 (N=3624 axons)
KDE
Mode (KDE): 0.38 µm
90th percentile: 0.73 µm
Shrinkage Corrected (37.15%)
KDE
Mode (KDE): 0.61 µm
90th percentile: 1.17 µm
rat27.3 (N=33364 axons)
KDE
Mode (KDE): 0.27 µm
90th percentile: 0.71 µm
Shrinkage Corrected (37.15%)
KDE
Mode (KDE): 0.43 µm
90th percentile: 1.13 µm
rat28.2 (N=47683 axons)
KDE
Mode (KDE): 0.27 µm
90th percentile: 0.62 µm
Shrinkage Corrected (37.15%)
KDE
Mode (KDE): 0.44 µm
90th percentile: 0.99 µm
Density
Axon Diameter (µm)

Fig. S2. Overview of axon diameters from segmented TEM images from EPON-embedded CC-tissue of individual rats. A kernel density estimation (KDE) is applied to all diameter distributions of segmented axons. Mode values were obtained from the peak of the KDE. The shrinkage correction is based on the average of the calculated group mode shrinkage and shrinkage of the 90th percentile – from the Epon-TEM (Fig. S2) and Cryo-TEM (Fig. S3), respectively.

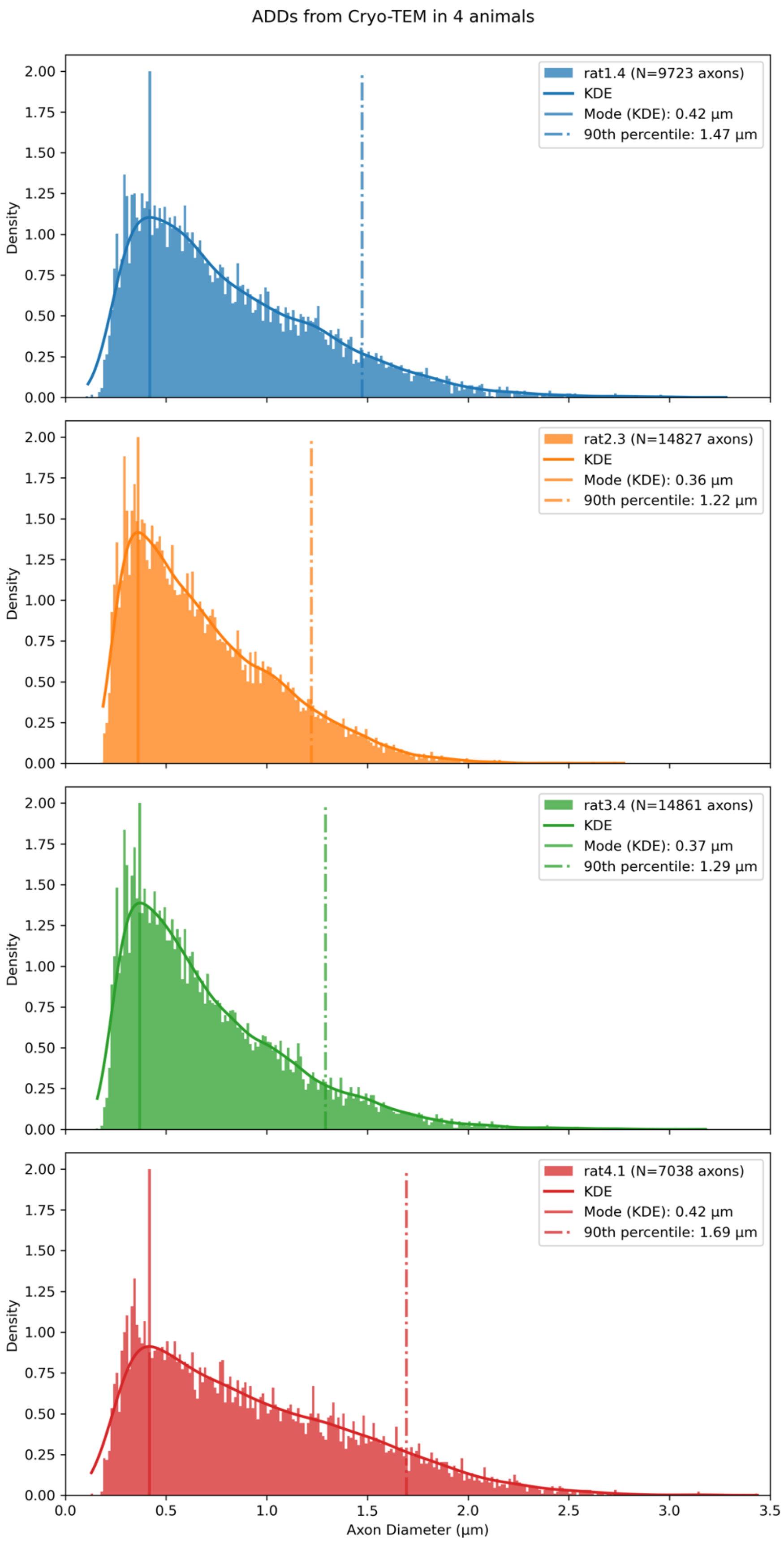
ADDs from Cryo-TEM in 4 animals
rat1.4 (N=9723 axons)
KDE
Mode (KDE): 0.42 µm
90th percentile: 1.47 µm
rat2.3 (N=14827 axons)
KDE
Mode (KDE): 0.36 µm
90th percentile: 1.22 µm
rat3.4 (N=14861 axons)
KDE
Mode (KDE): 0.37 µm
90th percentile: 1.29 µm
rat4.1 (N=7038 axons)
KDE
Mode (KDE): 0.42 µm
90th percentile: 1.69 µm
Density
Axon Diameter (µm)

Fig. S3. Overview of axon diameters from segmented TEM images from Cryo-embedded CC-tissue of individual rats. A kernel density estimation (KDE) is applied to all diameter distributions of segmented axons. Mode values were obtained from the peak of the KDE. The shrinkage correction applied in previous image (Fig. S2) is based on the shrinkage from the mode and the 90th percentile – comparing the distributions from the Epon-TEM and the Cryo-TEM.

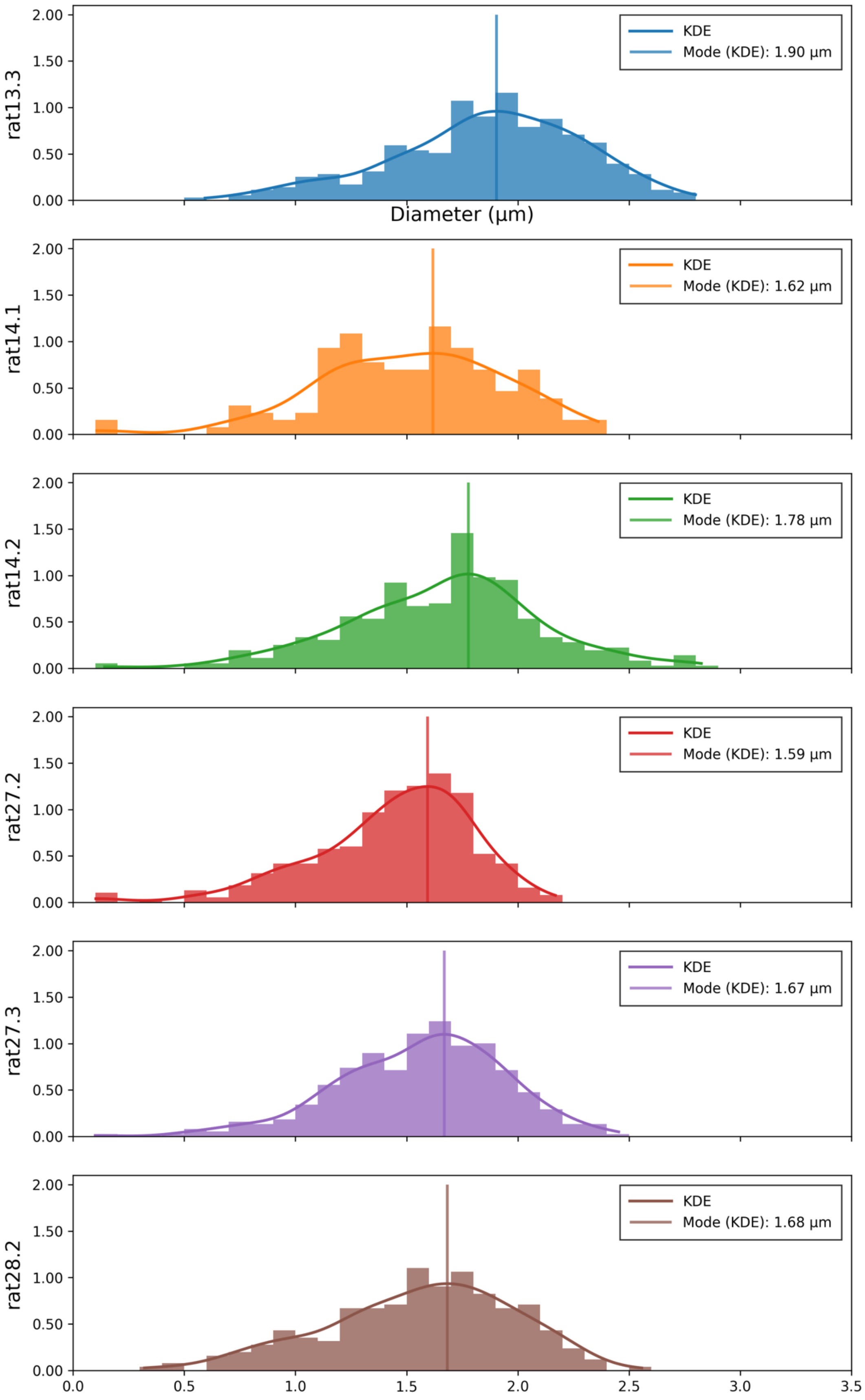

rat13.3
KDE
Mode (KDE): 1.90 μm
Diameter (μm)
rat14.1
KDE
Mode (KDE): 1.62 μm
rat14.2
KDE
Mode (KDE): 1.78 μm
rat27.2
KDE
Mode (KDE): 1.59 μm
rat27.3
KDE
Mode (KDE): 1.67 μm
rat28.2
KDE
Mode (KDE): 1.68 μm
0.00
0.50
1.00
1.50
2.00
0.0
0.5
1.0
1.5
2.0
2.5
3.0
3.5

Fig. S4: Overview of predictions of axon diameters from dw-MRI of individual rats. N=number of extracted voxels, defined by the tractography streamlines projecting between the bilateral M1s and passing through the midsagittal plane. A kernel density estimation (KDE) is applied to all diameter distributions of the estimated axon diameters from dw-MRI. Mode values were obtained from the peak of the KDE.

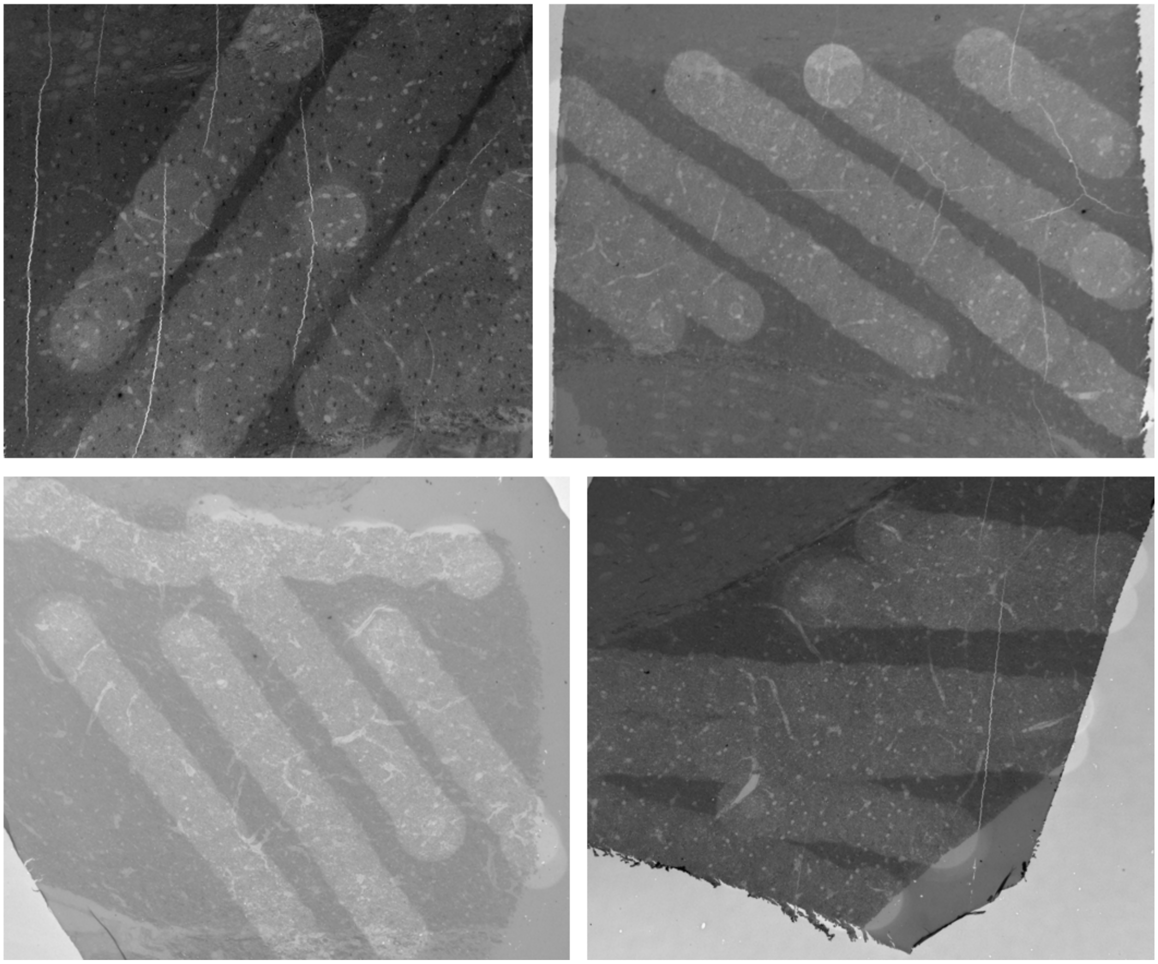

Fig. S5: TEM overview images at low magnification of the acquisition traverse paths at high magnification (4200x) from four rats. The overall samples correspond to ultrathin sections of the Ø=1mm punctures. The darker band in the middle of the sample corresponds to the corpus callosum. Each bleached circle corresponds to a focus area for image acquisition. The focus bleached circles are larger than the actual square field of view (2048x2048 pixels) and care was taken to avoid overlap of the images. The images were acquired in an unbiased manner by an other-wise uninvolved TEM-professional.

Table S1. Latencies from P1 and N1 for all animals

Animal	P1 Peak				N1 Onset				N1 Peak		
	**Median (ms)**	**SD (ms)**	**Vals (N)**		**Median (ms)**	**SD (ms)**	**Vals (N)**		**Median (ms)**	**SD (ms)**	**Vals (N)**
rat27.1	4.83	0.10	2		6.03	0.42	9		10.27	0.44	9
rat27.2	5.87	0.27	22		7.63	0.36	24		11.90	0.50	24
rat27.3	4.30	0.12	19		5.57	0.18	23		9.30	0.30	23
rat28.2	7.07	0.42	12		8.94	0.44	12		13.62	0.62	12
rat33.1	-	-	0		-	-	0		-	-	0
rat35.2	3.30	0.21	3		4.73	0.35	15		10.93	0.53	15
rat35.3	6.03	0.39	11		8.83	0.44	11		12.70	0.62	11
rat35.4	7.42	0.08	2		10.52	0.15	2		15.68	0.35	2
rat9.4	-	-	0		-	-	0		-	-	0
rat10.1	5.40	0.22	7		6.60	0.29	8		10.79	0.76	8
rat13.2	-	-	0		5.05	0.34	10		9.62	0.37	10
rat13.3	7.32	0.34	4		8.87	0.28	4		14.18	0.77	4
rat14.1	3.40	0.26	3		5.03	0.51	15		9.93	0.49	15
rat14.2	3.57	0.25	4		5.87	0.28	22		10.45	0.43	22
rat14.3	6.87	0.39	16		8.90	0.36	16		12.98	0.75	16
rat14.5	6.98	0.32	14		8.58	0.28	14		12.87	0.66	14
**median**	**5.87**		**13**		**7.12**		**14**		**11.42**		**14**
**Mean ± Mean SD (within)**	**5.57**	**0.26**	**13**		**7.22**	**0.33**	**14**		**11.80**	**0.54**	**14**
**Mean ± SEM**	**5.57**	**0.43**	**13**		**7.22**	**0.50**	**14**		**11.80**	**0.51**	**14**
**Mean + SD (Between)**	**5.57**	**1.49**	**13**		**7.22**	**1.81**	**14**		**11.80**	**1.84**	**14**

Table S2. Electrode and fiber depths measured from T2 FISP 3D MRI

RatID	ScanID	Electrode Depth (left) (mm)	Fiber Depth (right) (mm)
rat33.1	M0613	-0.97	-0.97
rat13.2	M0642	-1.10	-1.22
rat13.3	M0649	-1.01	-1.33
rat14.1	M0620	-1.07	-1.23
rat14.2	M0641	-1.07	-0.94

Table S3. Transcallosal pathway length between bilateral M1s, from tractography of dw-MRI. The total length is the sum of the left and right tractogram – including potential adjustments. The vertical height of the tractogram in cortex, before it projects into Corpus Callosum, had to be adjusted in one subject ("Adjust H"). This was likely due to tissue damage from the electrode, requiring seeding further away from the electrode depth. Corpus Callosum was chosen as target and termination ROI and the width of the ROI ("Adjust mid CC") is thus not included in either tractogram and had to be added subsequently.

RatID	ScanID	Left Tract (mm)	Right Tract (mm)	Adjust H (mm)	Adjust mid CC (mm)	Total (mm)
rat27.1	M0761	-	-	-	-	-
rat27.2	M0781	5.39±0.73	4.61±0.67	0	0.23	10.23
rat27.3	M0783	5.07±0.83	5.15±0.63	0	0.23	10.45
rat28.2	M0787	5.74±1.59	6.83±2.23	0	0.23	12.80
rat33.1	M0613	4.98±1.16	4.45±0.80	0	0.23	9.66
rat13.2	M0642	7.58±2.40	5.70±2.23	0	0.23	13.51
rat13.3	M0751	3.52±0.76	6.54±1.81	1.012	0.23	11.30
rat14.1	M0765	5.64±1.53	6.46±1.87	0	0.23	12.33
rat14.2	M0773	5.50±1.26	5.78±1.29	0	0.23	11.51
**AVG**						**11.47 ± 0.47**

Table S4. Non-exhaustive overview of histological investigations of axon diameter distribution in Corpus Callosum of Rats.
Abbreviations: M/F: Male / Female; P150: Postnatal day 150, CC: Corpus Callosum, TEM: Transmission Electron Microscopy.

Sex	Age	Region	Value (µm)	Metric	Imaging	Fixation and Tissue State	Study
M	P150	Anterior CC<br>Middle CC<br>Poterior	~0.58<br>~0.56<br>~0.50	mean	TEM<br>16,500x	Perfusion fixation:<br>4% Formaldehyde + 1% Gluteraldehyde,<br>State: Dehydrated, Resin-embedded	(Salas-Lucia et al., 2020)
M	P58	Forceps Minor	~0.89	mean	LM<br>100x (oil)	Perfusion fixation:<br>1% Formaldehyde + 1.25% Gluteraldehyde,<br>State: Dehydrated, Resin-embedded	(McDougall et al., 2018)
M	P120	#1: Genu<br>#2<br>#3: Midbody<br>#4<br>#5: Splenium	~0.9<br>~0.6<br>~1.0<br>~1.1<br>~0.6	mode<br>(read from axon diameter distributions)	TEM<br>15,000x	Perfusion fixation:<br>Formaldehyde + Gluteraldehyde<br>Post fixation: 2.5% Gluteraldehyde<br>State: Dehydrated, Resin-embedded	(Barazany et al., 2009)
M + F	P70	Splenium	~0.5	mean	TEM<br>20,000x	Perfusion fixation:<br>1% Formaldehyde + 1.0% Gluteraldehyde.<br>Post fixation:<br>2% Formaldehyde + 0.1% Gluteraldehyde.<br>State: Dehydrated, Resin-embedded	(Pesaresi et al., 2015)
F	P84	Genu	~1.22	median	Confocal LM, 63x	Perfusion fixation: 4% Formaldehyde<br>Post fixation: 4% Formaldehyde<br>State: Hydrated	(Veraart et al., 2020)
M+F	Young adult	Splenium	[0.11 - 0.2]	modal bin<br>(binned interval)	TEM<br>33,000x	Perfusion fixation: 4% Gluteraldehyde<br>Post fixation: 4% Gluteraldehyde<br>State: Dehydrated, Resin-embedded	(Olivares et al., 2001)

Table S5. **Overview of subjects and which modalities they provide data to.**

RatID	Opto-ElPhys	MRI			Microscopy			Notes
		Tract	Diameter	Struct	F-LM	Epon-TEM	Cryo-TEM	
rat27.1	X	(X)	(X)	-	-	-	-	Tractography was unsuccessful and in turn unsuccessful extraction from DiameterMaps
rat27.2	X	X	X	-	-	(X)	-	Sample prep. for TEM was unsuccessful
rat27.3	X	X	X	-	-	X	-	
rat28.2	X	X	X	-	-	X	-	
rat33.1	(X)	X	(X)	X	-	-	-	Poor OptoElPhys response<br>Unsuccesful fitting of DiameterMaps
rat35.2	X	-	-	-	-	-	-	
rat35.3	X	-	-	-	-	-	-	
rat35.4	X	-	-	-	-	-	(X)	Sample prep. for TEM was unsuccessful
rat04.2	-	-	-	-	-	-	(X)	Discarded, as methods varied too much, compared to subsequent Cryo-TEM rats.
rat09.4	(X)	-	-	-	(X)	-	-	No *robust* OptoElPhys peaks
rat10.1	X	-	-	-	(X)	-	-	
rat13.2	(X)	X	(X)	X	-	(X)	-	No *robust* OptoElPhys peaks<br>Sample prep. for TEM was unsuccessful<br>Unsuccesful fitting of DiameterMaps
rat13.3	X	X	X	X	-	-	-	
rat14.1	X	X	X	X	-	X	-	
rat14.2	X	X	X	X	-	X	-	
rat14.3	X	-	-	-	-	-	-	
rat14.5	X	-	-	-	X	X	-	
rat15.1	-	-	-	-	-	-	(X)	Sample prep. for TEM was unsuccessful
rat01.4	-	-	-	-	-	-	X	
rat02.3	-	-	-	-	-	-	X	
rat03.4	-	-	-	-	-	-	X	
rat04.1	-	-	-	-	-	-	X	
Total (22)	13 (+3)	8 (+1)	7(+2)	5	1(+2)	5 (+2)	4 (+3)	

SI References

Alexander, D. C., Hubbard, P. L., Hall, M. G., Moore, E. A., Ptito, M., Parker, G. J. M., & Dyrby, T. B. (2010). Orientationally invariant indices of axon diameter and density from diffusion MRI. *NeuroImage*, *52*(4), 1374–1389. https://doi.org/10.1016/j.neuroimage.2010.05.043

Andersson, M., Kjer, H. M., Rafael-Patino, J., Pacureanu, A., Pakkenberg, B., Thiran, J.-P., Ptito, M., Bech, M., Bjorholm Dahl, A., Andersen Dahl, V., & Dyrby, T. B. (2020). Axon morphology is modulated by the local environment and impacts the noninvasive investigation of its structure-function relationship. *Proceedings of the National Academy of Sciences of the United States of America*, *117*(52), 33649–33659. https://doi.org/10.1073/pnas.2012533117

Andersson, M., Pizzolato, M., Kjer, H. M., Skodborg, K. F., Lundell, H., & Dyrby, T. B. (2022). Does powder averaging remove dispersion bias in diffusion MRI diameter estimates within real 3D axonal architectures? *NeuroImage*, *248*, 118718. https://doi.org/10.1016/j.neuroimage.2021.118718

Bak & Nielsen. (1997). REPULSION, A novel approach to efficient powder averaging in solid-state NMR. *Journal of Magnetic Resonance (San Diego, Calif. : 1997)*, *125*(1), 132–139. https://doi.org/10.1006/jmre.1996.1087

Barazany, D., Basser, P. J., & Assaf, Y. (2009). In vivo measurement of axon diameter distribution in the corpus callosum of rat brain. *Brain : A Journal of Neurology*, *132*(Pt 5), 1210–1220. https://doi.org/10.1093/brain/awp042

Burcaw, L. M., Fieremans, E., & Novikov, D. S. (2015). Mesoscopic structure of neuronal tracts from time-dependent diffusion. *NeuroImage*, *114*, 18–37. https://doi.org/10.1016/j.neuroimage.2015.03.061

Caminiti, R., Carducci, F., Piervincenzi, C., Battaglia-Mayer, A., Confalone, G., Visco-Comandini, F., Pantano, P., & Innocenti, G. M. (2013). Diameter, length, speed, and conduction delay of callosal axons in macaque monkeys and humans: Comparing data from histology and magnetic resonance imaging diffusion tractography. *The Journal of Neuroscience : The Official Journal of the Society for Neuroscience*, *33*(36), 14501–14511. https://doi.org/10.1523/JNEUROSCI.0761-13.2013

Caminiti, R., Ghaziri, H., Galuske, R., Hof, P. R., & Innocenti, G. M. (2009). Evolution amplified processing with temporally dispersed slow neuronal connectivity in primates. *Proceedings of the National Academy of Sciences of the United States of America*, *106*(46), 19551–19556. https://doi.org/10.1073/pnas.0907655106

Dyrby, T. B., Baaré, W. F. C., Alexander, D. C., Jelsing, J., Garde, E., & Søgaard, L. V. (2011). An ex vivo imaging pipeline for producing high-quality and high-resolution diffusion-weighted imaging datasets. *Human Brain Mapping*, *32*(4), 544–563. https://doi.org/10.1002/hbm.21043

Dyrby, T. B., Innocenti, G. M., Bech, M., & Lundell, H. (2018). Validation strategies for the interpretation of microstructure imaging using diffusion MRI. *NeuroImage*, *182*, 62–79. https://doi.org/10.1016/j.neuroimage.2018.06.049

Fan, Q., Nummenmaa, A., Witzel, T., Ohringer, N., Tian, Q., Setsompop, K., Klawiter, E. C., Rosen, B. R., Wald, L. L., & Huang, S. Y. (2020). Axon diameter index estimation independent of fiber orientation distribution using high-gradient diffusion MRI. *NeuroImage*, *222*, 117197. https://doi.org/10.1016/j.neuroimage.2020.117197

Hursh, J. B. (1939). Conduction velocity and diameter of nerve fibers. *Am J Physiol*, *127*, 131–139. https://doi.org/10.1152/ajplegacy.1939.127.1.131

Jones, D. K., Horsfield, M. A., & Simmons, A. (1999). Optimal strategies for measuring diffusion in anisotropic systems by magnetic resonance imaging. *Magnetic Resonance in Medicine*, *42*(3), 515–525.

Kaden, E., Kruggel, F., & Alexander, D. C. (2016). Quantitative mapping of the per-axon diffusion coefficients in brain white matter: Quantitative Mapping of the Per-Axon Diffusion Coefficients. *Magnetic Resonance in Medicine*, *75*(4), 1752–1763. https://doi.org/10.1002/mrm.25734

Kaur, S., Lazar, R., & Metherate, R. (2004). Intracortical pathways determine breadth of subthreshold frequency receptive fields in primary auditory cortex. *Journal of Neurophysiology*, *91*(6), 2551–2567. https://doi.org/10.1152/jn.01121.2003

Kellner, E., Dhital, B., Kiselev, V. G., & Reisert, M. (2016). Gibbs-ringing artifact removal based on local subvoxel-shifts. *Magnetic Resonance in Medicine*, *76*(5), 1574–1581. https://doi.org/10.1002/mrm.26054

Kroenke, C. D., Ackerman, J. J. H., & Yablonskiy, D. A. (2004). On the nature of the NAA diffusion attenuated MR signal in the central nervous system. *Magnetic Resonance in Medicine*, *52*(5), 1052–1059. https://doi.org/10.1002/mrm.20260

Lee, H.-H., Jespersen, S. N., Fieremans, E., & Novikov, D. S. (2020). The impact of realistic axonal shape on axon diameter estimation using diffusion MRI. *NeuroImage*, *223*, 117228. https://doi.org/10.1016/j.neuroimage.2020.117228

Ma, X., Uğurbil, K., & Wu, X. (2020). Denoise magnitude diffusion magnetic resonance images via variance-stabilizing transformation and optimal singular-value manipulation. *NeuroImage*, *215*, 116852. https://doi.org/10.1016/j.neuroimage.2020.116852

Makarov, V. A., Schmidt, K. E., Castellanos, N. P., Lopez-Aguado, L., & Innocenti, G. M. (2008). Stimulus-dependent interaction between the visual areas 17 and 18 of the 2 hemispheres of the ferret (Mustela putorius). *Cerebral Cortex (New York, N.Y.: 1991)*, *18*(8), 1951–1960. https://doi.org/10.1093/cercor/bhm222

McDougall, S., Vargas Riad, W., Silva-Gotay, A., Tavares, E. R., Harpalani, D., Li, G.-L., & Richardson, H. N. (2018). Myelination of axons corresponds with faster transmission speed in the prefrontal cortex of developing male rats. *eNeuro*, *5*(4). https://doi.org/10.1523/ENEURO.0203-18.2018

Olivares, R., Montiel, J., & Aboitiz, F. (2001). Species differences and similarities in the fine structure of the mammalian corpus callosum. *Brain, Behavior and Evolution*, *57*(2), 98–105. https://doi.org/10.1159/000047229

P. A. Cook, Y. Bai, S. N.-G., K. K. Seunarine, M. G. Hall, G. J. Parker, D. C. Alexander. (2006). Camino: Open-source diffusion-MRI reconstruction and processing. *Proc. Intl. Soc. Mag. Reson. Med.*, *14*, 2759. http://www.cs.ucl.ac.uk/research/medic/camino/files/camino_2006_abstract.pdf

Packer, K. J., & Rees, C. (1972). Pulsed NMR studies of restricted diffusion. I. Droplet size distributions in emulsions. *Journal of Colloid and Interface Science*, *40*(2), 206–218. https://doi.org/10.1016/0021-9797(72)90010-0

Pesaresi, M., Soon-Shiong, R., French, L., Kaplan, D. R., Miller, F. D., & Paus, T. (2015). Axon diameter and axonal transport: In vivo and in vitro effects of androgens. *NeuroImage*, *115*, 191–201. https://doi.org/10.1016/j.neuroimage.2015.04.048

Preibisch, S., Saalfeld, S., & Tomancak, P. (2009). Globally optimal stitching of tiled 3D microscopic image acquisitions. *Bioinformatics (Oxford, England)*, *25*(11), 1463–1465. https://doi.org/10.1093/bioinformatics/btp184

Riise, J., & Pakkenberg, B. (2011). Stereological estimation of the total number of myelinated callosal fibers in human subjects. *Journal of Anatomy*, *218*(3), 277–284. https://doi.org/10.1111/j.1469-7580.2010.01333.x

Salas-Lucia, F., Pacheco-Torres, J., González-Granero, S., García-Verdugo, J. M., & Berbel, P. (2020). Transient hypothyroidism during lactation alters the development of the corpus callosum in rats. An , javax.xml.bind.JAXBElement@3d463821, magnetic resonance image and electron microscopy study. *Frontiers in Neuroanatomy*, *14*, 33. https://doi.org/10.3389/fnana.2020.00033

Skoven, C. S., Tomasevic, L., Kvitsiani, D., Pakkenberg, B., Dyrby, T. B., & Siebner, H. R. (2022). Dose-response relationship between the variables of unilateral optogenetic stimulation and transcallosal evoked responses in rat motor cortex. *Frontiers in Neuroscience*, *16*, 968839. https://doi.org/10.3389/fnins.2022.968839

Tettoni, L., Lehmann, P., Houzel, J. C., & Innocenti, G. M. (1996). Maxsim, software for the analysis of multiple axonal arbors and their simulated activation. *Journal of Neuroscience Methods*, *67*(1), 1–9. https://doi.org/10.1016/0165-0270(95)00095-x

Tomasi, S., Caminiti, R., & Innocenti, G. M. (2012). Areal differences in diameter and length of corticofugal projections. *Cerebral Cortex (New York, N.Y. : 1991)*, *22*(6), 1463–1472. https://doi.org/10.1093/cercor/bhs011

Tournier, J.-D., Smith, R., Raffelt, D., Tabbara, R., Dhollander, T., Pietsch, M., Christiaens, D., Jeurissen, B., Yeh, C.-H., & Connelly, A. (2019). MRtrix3: A fast, flexible and open software framework for medical image processing and visualisation. *NeuroImage*, *202*, 116137. https://doi.org/10.1016/j.neuroimage.2019.116137

van Gelderen, P., Despres, D., Vanzijl, P. C. M., & Moonen, C. T. W. (1994). Evaluation of Restricted Diffusion in Cylinders. Phosphocreatine in Rabbit Leg Muscle. *Journal of Magnetic Resonance, Series B*, *103*(3), 255–260. https://doi.org/10.1006/jmrb.1994.1038

Veraart, J., Nunes, D., Rudrapatna, U., Fieremans, E., Jones, D. K., Novikov, D. S., & Shemesh, N. (2020). Nonivasive quantification of axon radii using diffusion MRI. *eLife*, *9*. https://doi.org/10.7554/eLife.49855

Waxman, S. G., & Bennett, M. V. (1972). Relative conduction velocities of small myelinated and non-myelinated fibres in the central nervous system. *Nature: New Biology*, *238*(85), 217–219. https://doi.org/10.1038/newbio238217a0

Yushkevich, P. A., Piven, J., Hazlett, H. C., Smith, R. G., Ho, S., Gee, J. C., & Gerig, G. (2006). User-guided 3D active contour segmentation of anatomical structures: Significantly improved efficiency and reliability. *NeuroImage*, *31*(3), 1116–1128. https://doi.org/10.1016/j.neuroimage.2006.01.015

Zaimi, A., Wabartha, M., Herman, V., Antonsanti, P.-L., Perone, C. S., & Cohen-Adad, J. (2018). AxonDeepSeg: Automatic axon and myelin segmentation from microscopy data using convolutional neural networks. *Scientific Reports*, *8*(1), 3816. https://doi.org/10.1038/s41598-018-22181-4
